# The Effects of Biome Stability During the Quaternary on Plant Diversity

**DOI:** 10.1002/ece3.73884

**Published:** 2026-06-30

**Authors:** Simon Scheiter, Julia Brugger, Thomas Hickler

**Affiliations:** ^1^ Senckenberg Biodiversity and Climate Research Centre (SBiK‐F) Frankfurt am Main Germany; ^2^ Geo‐ and Environmental Research Center University of Tübingen Tübingen Germany; ^3^ University of Tübingen, Cluster of Excellence (EXC 3121): TERRA—Terrestrial Geo‐Biosphere Interactions in a Changing World Tübingen Germany; ^4^ Institute of Physical Geography Goethe University Frankfurt am Main Frankfurt am Main Germany

**Keywords:** bioclimatic variables, biome stability, biomes, quaternary, species distribution model, species richness

## Abstract

The distribution of global biomes has changed during Earth history in response to changes in the climate conditions. We aimed to study how climate change during the Quaternary, a period characterized by periodic transitions between glacials and interglacials and a cooling trend, influenced the expansion of biomes and biome stability. We further studied the hypothesis that biome stability can explain current patterns of plant species richness. We used machine learning and correlative models, current bioclimatic conditions, and an observation‐based biome map to model the current global biome distribution. Then, we drove the model with bioclimatic conditions for the last 2.6 million years at a 1000 year temporal resolution to obtain global climate‐driven biome suitability maps for this period. Our results indicate that during the study period, biomes were stable in large parts of tropical forests, deserts, boreal and temperate forests, while northern Europe, northern and central America were core areas of biome change. Biome shifts in the far North were caused by regular transitions between boreal vegetation and ice. We analyzed the relation between biome stability and current plant species richness. In tropical rainforests, species richness was positively related to biome stability, but globally the overlap between stable biomes and centers of high species richness was weak. Our modeling framework provides important insights into the stability of biomes during the Quaternary. We suggest that biome stability cannot fully explain species richness, indicating that other factors such as fire or spatial heterogeneity are important. Even though our approach is based on climate only, it is appropriate for further studies due to its general applicability to and spatial and temporal resolution and high computational performance.

## Graphical abstract

We used machine learning to reconstruct global biome changes over the last 2.6 million years, revealing that tropical forests, deserts, and temperate forests were relatively stable, whereas northern Europe and North America experienced frequent biome shifts due to ice sheet expansions. Although biome stability in tropical rainforests correlated with higher plant species richness, stable biomes did not consistently align with areas of high biodiversity globally. This finding indicates that additional factors influenced current species richness patterns.

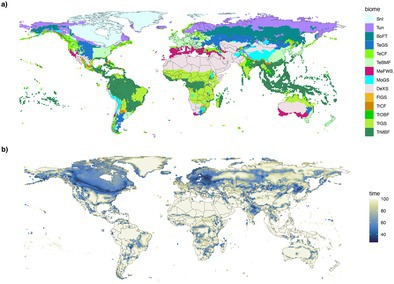

## 1 Introduction

The Quaternary spans from around 2.6 million years ago to now and is characterized by regular glacial and interglacial periods (Oschmann 2018). Those cycles were initiated towards the end of the Pliocene due to the closure of the Isthmus of Panama, that is the connection of previously separated North and South America (Haug and Tiedemann 1998; Bartoli et al. 2005). As a consequence, ocean and atmospheric circulations changed which facilitated the glaciation of the Northern hemisphere (Bartoli et al. 2005). The Quaternary is further characterized by a long‐term trend of decreasing temperatures that has persisted throughout the Cenozoic, and changes in the periodicity of glaciations to transition from a 41 ka period to a 100 ka period around 800 ka ago. Multiple hypotheses have been proposed to explain this Middle Pleistocene transition, but a final explanation remains open (Miller et al. 2010; Legrain et al. 2023). Such changes in the climate had substantial impacts on the global distribution of terrestrial biomes. Empirical evidence, for instance from pollen records, clearly shows biome changes during the Quaternary (e.g., Harrison 2017). It has been hypothesized that stability of past climate and biomes may explain current patterns of species richness (e.g., Sandel et al. 2011; Huntley et al. 2016). For example, climate and biome stability at paleo‐ecological time scales may reduce species extinction rates compared to regions with frequent biome changes, and thereby maintain species richness (Brown et al. 2020; Huntley et al. 2021). Areas with climate stability and low climate velocity between glacial and interglacial stages may have acted as refugia for species with low dispersal potential, endemics or specialists (Sandel et al. 2011). Huntley et al. (2016) and Colville et al. (2020) showed, that biome stability is a better predictor of diversity patterns in South Africa than climate stability. Alternative hypotheses such as the “species pump” mechanism (Li and Li 2018; Dixit et al. 2023) suggest that Pleistocene sea‐level fluctuations and complex topography promoted diversification and species richness by allowing speciation in separated populations during periods of high sea‐level, and reassembly of species during periods of low sea‐level.

Testing these hypotheses requires knowledge on past biome distributions. Such knowledge can be derived from a suite of paleo‐ecological proxy data indicating the presence of biomes under past climate conditions (e.g., BIOME6000 or LegacyPollen2.0, Harrison 2017; Li et al. 2024). Such data are, however, constrained by the number of sites where data can be recorded, and by the time periods represented by proxy data. To extrapolate in space and time, both correlative and machine learning approaches as well as process‐based models have been used. Correlative and machine learning models, including species distribution models (SDMs; Franklin 2009) are widely used to model species distributions under current and future climate conditions (e.g., Dyderski et al. 2018). SDMs have also been used to model biomes (Heubes et al. 2011; Midgley et al. 2003) and to reconstruct species distributions in paleo‐ecological studies. Yet, previous studies were often conducted for specific regions, species and time periods such as the LGM (e.g., Potts et al. 2013; Chiarenza et al. 2023) instead of the Quaternary at the global scale. Allen et al. (2020) and Huntley et al. (2021) modeled global biomes during the last 140 ka and 800 ka, respectively, using the process‐based LPJ‐GUESS dynamic global vegetation model (DGVM; Smith et al. 2014). Zeller et al. (2023) used climate data simulated by the Community Earth System Model (CESM) and the BIOME4 dynamic vegetation model to model biome patterns for the last 3 million years and to study how biome changes influenced hominin expansion during that period.

Recently, Barreto et al. (2023) presented bioclimatic variables for the last 5 million years with a temporal resolution of 1000 years at the global scale. Bioclimatic variables represent means, variability, and seasonality of precipitation and temperature for given time periods (Booth et al. 2014). We made use of these data to model climate‐driven global biome suitability maps for the last 2.6 million years. Therefore, we first developed correlative and machine learning models informed by bioclimatic variables for current conditions to reproduce an observation‐based biome map (Olson et al. 2001). Then we drove the models for past bioclimatic conditions to obtain global biogeographic patterns of biomes for the Quaternary. Accordingly, our results should be interpreted as first‐order, climate‐driven constraints on biome distributions rather than predictions or reconstructions of realized vegetation patterns. Our correlative and machine learning approach is computationally efficient and allows us to generate biome maps for large temporal and spatial scales, and high agreement with current observation‐based biome maps, even though globally important disturbances, in particular fire, which is commonly implemented in DGVMs, are only accounted for indirectly through the climatic drivers of fuel dynamics and wildfires.

Using this modeled time series of biome suitability maps for the Quaternary, we asked (1) how did the biome suitability distributions change during this period, given changes in bioclimatic conditions? (2) Where are core areas of biome change and biome stability? (3) Do areas with biome stability overlap with current hotspots of vascular plant species richness?

## 2 Materials and Methods

### 2.1 Biome Modeling

We used bioclimatic variables provided by Barreto et al. (2023, PALEO‐PGEM‐Series). This data set provides 17 of the 19 common bioclimatic variables (Booth et al. 2014) for the last 5 million years until 1950 at 1° spatial resolution and 1000 years temporal resolution. These variables describe mean, variability and seasonality of precipitation and temperature (see Table S1), and they can be derived from monthly precipitation and temperature time series for a given time period. To obtain the bioclimatic variables, Barreto et al. (2023) created emulations using the intermediate‐complexity atmosphere‐ocean general circulation model (PALEO‐PGEM) and simulation results from the PLASIM‐GENIE GCM. Subsequently, the emulations were downscaled and anomalies were adjusted using the CHELSA climate data (Karger et al. 2017) for current conditions. We used the time slices of the last 2.6 million years (i.e., the Quaternary) for our species distribution modeling.

To create biome suitability maps for the Quaternary, correlative and machine learning models commonly applied for species distribution modeling (SDMs; Franklin 2009) were used. Those models correlate biogeographic patterns of species with bioclimatic variables, while other environmental variables such as CO_2_, radiation or soil, and processes such as competition or fire are ignored or only implicitly considered (e.g., fire is to a large extent driven by climate). We first created a model for current climate conditions using the bioclimatic variables for 1950 as included in Barreto et al. (2023) and the Olson et al. (2001) biome map provided by Fischer et al. (2022). We aggregated the Olson et al. (2001) biome map from the 10 × 10 km spatial resolution to the 1° resolution of the bioclimatic data (Barreto et al. 2023) using the “terra” R package (Hijmans et al. 2026). As the biome maps are categorical, we used the nearest neighbor method for aggregation. We removed biomes that occur in less than 40 grid cells and grid cells not covered by vegetation such as water and built‐up areas. The Olson et al. (2001) biome map was used because it provides potential natural vegetation derived from expert‐based biogeographical zonation and species distributions, and because it is often used as reference biome map for biome‐specific analyses or by international initiatives such as IPBES or WWF. We include ice‐covered areas (“Snow and ice” in Olson et al. 2001) as a functional category to capture climate‐driven exclusion of vegetation, although we acknowledge that ice dynamics are governed by processes not explicitly represented in our model. For the modeling, we selected six of the 17 bioclimatic variables by two criteria. First, we included mean annual temperature (bio1) and mean annual precipitation (bio12) as those variables are commonly used to represent the bioclimatic niche of species or biomes (e.g., Whittaker 1975). Second, we selected uncorrelated variables (correlation < 0.5) from the remaining variables. These were mean temperature of wettest quarter (bio8), mean temperature of driest quarter (bio9), precipitation of driest month (bio14), and precipitation of warmest quarter (bio18).

To create the biome models for current conditions, each biome type in the Olson et al. (2001) map was first modeled individually by using the coverage of the target biome as presence data. From the remaining area not covered by the target biome, 3 sets of 3500 background data points were randomly sampled for the modeling, to inform the models about the entire climatic space and climate conditions that are unsuitable for the target biome. Data were randomly split into training data and test data (80% and 20%, respectively) for model evaluation. Evaluation was repeated twice. Six different modeling approaches were applied: logistic regression (generalized linear models, GLM), classification tree analysis (CTA), artificial neural networks (ANN), surface range envelop (SRE), flexible discriminant analysis (FDA), and random forests (RF). Overall, an ensemble of 36 models was fitted per biome.

From this ensemble of all 36 models, a subset of models with TSS > 0.5 (True Skill Statistic) was selected and combined to an ensemble model by calculating the mean suitability of the remaining models. By evaluating the ensemble model with the bioclimatic variables for current conditions, we derived global suitability maps for each biome. The mean suitability of different biomes derived from averaging models with TSS > 0.5 is a continuous variable. Suitable areas can overlap, that is, suitability values of multiple biomes can be high in a grid cell and different biome types are supported by the prevailing bioclimatic conditions. To create a categorical biome map with only one biome type per grid cell, the biome type with the highest suitability value was identified for each grid cell and assigned. Suitability values derived from different algorithms are not necessarily comparable in a probabilistic sense; thus, the “winner‐takes‐all” classification should be interpreted as a heuristic assignment of dominant climate affinity rather than a strict likelihood‐based classification. Moreover, this approach simplifies the possibility of alternative states or biome mosaics within grid cells, which are known to occur in ecotonal regions (Pausas and Bond 2020). Systematic analyses of overlaps and alternative vegetation states are possible with our approach (Scheiter, Merkel, and Hickler 2025). The resulting biome map was compared to the Olson et al. (2001) biome map with the *κ* statistics (Monserud and Leemans 1992) to assess the performance of the modeling approach. Comparisons were conducted for maps including all biomes and for each biome individually. Therefore, we set the target biomes to present and all other biomes to absent and calculated the *κ* value for the resulting binary map. Models were fitted using the “biomod2” R package (Thuiller et al. 2026). Because the models were calibrated under present‐day climatic conditions, projections into the Quaternary inevitably involve extrapolation into non‐analogue climate spaces (Veloz et al. 2012). This may affect the reliability of biome suitability estimates in regions or periods where climatic combinations fall outside the contemporary range. Such calibration also applies to some extent to DGVMs (e.g., the bioclimatic limits still used in many models), but generally to a lesser extent than to purely correlative approaches like here.

Using the ensemble model fitted for current conditions, we then modeled biome patterns for each 1000 year time slice for past conditions, using the same approach that we applied for current conditions. Specifically, for each time slice, we created suitability maps for each biome and aggregated those maps to a categorical biome map by identifying the biome with the highest suitability. With this procedure, we created a time series of 2605 climate‐driven biome suitability maps for the last 2.6 Ma at 1000 years temporal resolution, starting in 1950.

### 2.2 Model Evaluation

To evaluate the performance of the model, modeled biome suitability maps were compared to the pollen‐derived biome reconstructions from BIOME6000 (Harrison 2017) and LegacyPollen2.0 (Li et al. 2024). BIOME6000 contains biome reconstructions for 0, 6 and 21 ka for 9117, 1819 and 291 sites, respectively. LegacyPollen2.0 contains reconstructions for multiple time periods for the last 21 ka. Similarly to BIOME6000, we selected reconstructions for 0, 6 and 21 ka from LegacyPollen2.0, where 2234, 1997 and 188 sites were available. For the model evaluation, modeled biome types were extracted for the grid cells that contain the sites of the reconstructions for the respective time period. As biome types in the pollen‐based data sets and the Olson et al. (2001) biome types did not match exactly, matching between biome types was necessary. For an objective, that is, quantitative and reproducible reclassification, we matched biomes such that the total number of matches was maximized (Table S2). As the BIOME6000 data included more biome types, we selected a matching biome in the Olson et al. (2001) map for each of the BIOME6000 biome types. Specifically, for each biome type in the BIOME6000 data set, we calculated the number of matches between the BIOME6000 biome and each of the Olson et al. (2001) biomes. Then, we selected the Olson et al. (2001) biome type with the highest number of matches. LegacyPollen2.0 contained less biome types than Olson et al. (2001), and we used the same approach to select a biome type in LegacyPollen2.0 for each Olson et al. (2001) biome (Table S3). This matching was conducted for the sites at 0 ka and then applied to the other time slices and to the Olson et al. (2001) biome map.

We extracted modeled biome types for long‐term pollen record sites provided by (Hooghiemstra et al. 2022). Many of those sites represent longer time periods than LegacyPollen2.0 and BIOME6000. After removing duplicate sites within the modeled 1° grid‐cells, 111 sites remained (Figure S1). For each site, we provide time series of the modeled biome type and the three most frequent biome types during our study period (Data S1, Figures S2–S4). These data provide a basis for future site‐specific comparisons and they allow for a qualitative assessment of biome stability over longer temporal scales.

### 2.3 Analyses

To quantify biome suitability change over time, the percent coverage of the land surface of each biome type was calculated for each time slice. The resulting time series were plotted using the “rioja” and the “riojaPlot” R packages (Juggins 2024, 2026). From these time series, we calculated minimum, mean, median, and maximum cover, the years when minimum and maximum cover were reached, as well as the absolute and relative difference between minimum and maximum cover. Histograms of biome coverage for the entire study period were created to test if distributions of biome coverage are uni‐modal or multi‐modal. Uni‐modal distributions indicate expansion and retreat of biomes around biome core areas, while multi‐modal distributions indicate large‐scale transitions between different biome types.

To study biome stability, we calculated for each grid cell and each biome type the percentage of time slices in which the respective grid cell was suitable to support the respective biome type. Values approaching 100% represent biome stability because a grid cell was suitable for the same biome for most of the time. The absence of a biome type with a high percent value of coverage represents a core area of biome change because the biome type changed during the considered time period. The biome type that covered each grid cell for most of the study period was identified. To study if biome stability changed during the Quaternary study period, we repeated the analysis for the first 150 ka and the last 150 ka of the study period. For both periods, the percent of time slices in which a grid cell was covered by the most frequent biome was calculated, and the change between both periods was calculated. We selected a 150 ka period because for the last period including the late Quaternary and the Holocene period, this period included the Eemian interglacial.

The relation between diversity and biome stability was analyzed by using the global map of vascular plant species richness (Cai et al. 2023). This map was created by machine learning and statistical methods based on inventories of around 300,000 species. While alternative maps of species richness are available (e.g., Sabatini et al. 2021), we selected the Cai et al. (2023) as it provides species richness at a regional scale instead of plot‐level scale, and the scale is more consistent with the 1° resolution of our model. The map was converted from the 30 arc second resolution to the 1° resolution of the modeled biome maps using the maximum value for aggregation. Then we identified centers of species richness. Following the definition of Cai et al. (2023), these are grid cells with more than 1765 species (90% percentile of species richness). In addition, we set the number to 1500 species to test the sensitivity of our results to the definition of centers of species richness. The spatial distribution of the centers of species richness was compared to the distribution of areas with biome stability. Here, we considered a grid cell as stable if it was covered by the same biome type for 100%, 95% or 90% of the study period. In contrast to the stability analyses that makes use of the entire time series, we only considered the last 150 ka of the study period for the stability‐species richness analyses. This approach assumes that current vegetation was primarily shaped by the last glacial cycles and less by the entire study period (Svenning and Skov 2007). If stable climate and stable biomes are causes of high species diversity, the spatial patterns of centers of diversity and biome stability are expected to overlap. Further, we plotted species richness against biome stability and fitted quantile regressions for the 5%, 50% and 95% percentiles, to avoid classification into stability and species richness hotspots. This analysis was conducted at global scale and separately per biome type under current climate conditions. To account for the latitudinal bias in the 1° grid used in our analysis, quantile regressions were weighted by the cosine of the latitude. This weighting ensures that each grid cell contributes to the model according to its actual area, preventing an over‐representation of smaller high‐latitude cells in the global and biome‐specific estimates.

Modeling and analyses were performed with R (R Core Team 2024). Plots were created using the “ggplot2” R package (Wickham 2016), animations of the biome distributions were created using the “gganimate” R package (Pedersen and Robinson 2025).

## 3 Results

### 3.1 Current and Past Biomes

**FIGURE 1:**
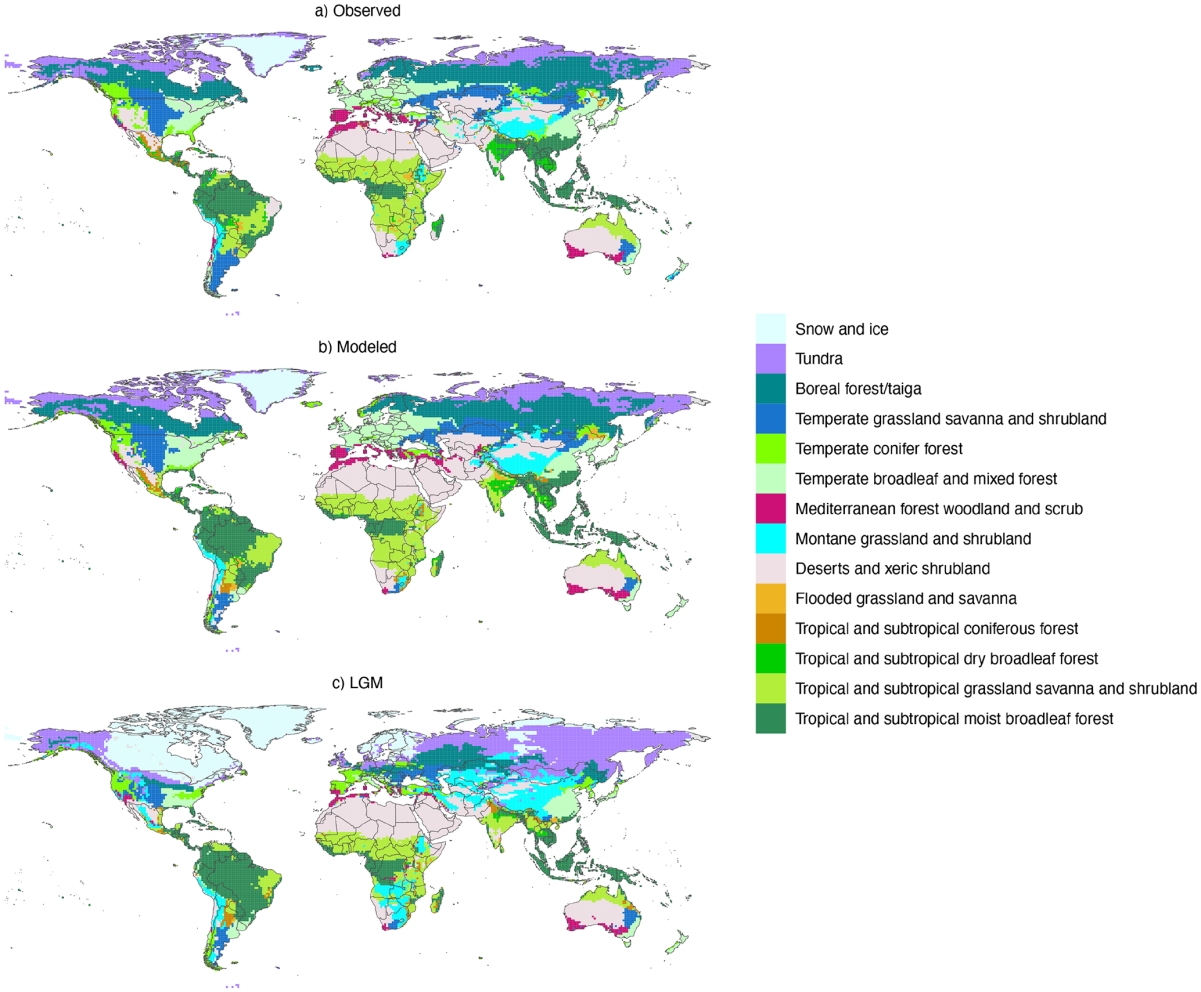
Observed, modeled current and modeled LGM biomes. (a) Observed biomes represent the Olson et al. (2001) biome map as included in the biome product compiled by Fischer et al. (2022). (b) Modeled biomes for current conditions were derived from machine learning and correlative models based on the Olson et al. (2001) biome map (see Section 2). (c) Modeled biomes for the LGM were derived from projecting biomes using the PGEM data (Barreto et al. 2023).

**FIGURE 2:**
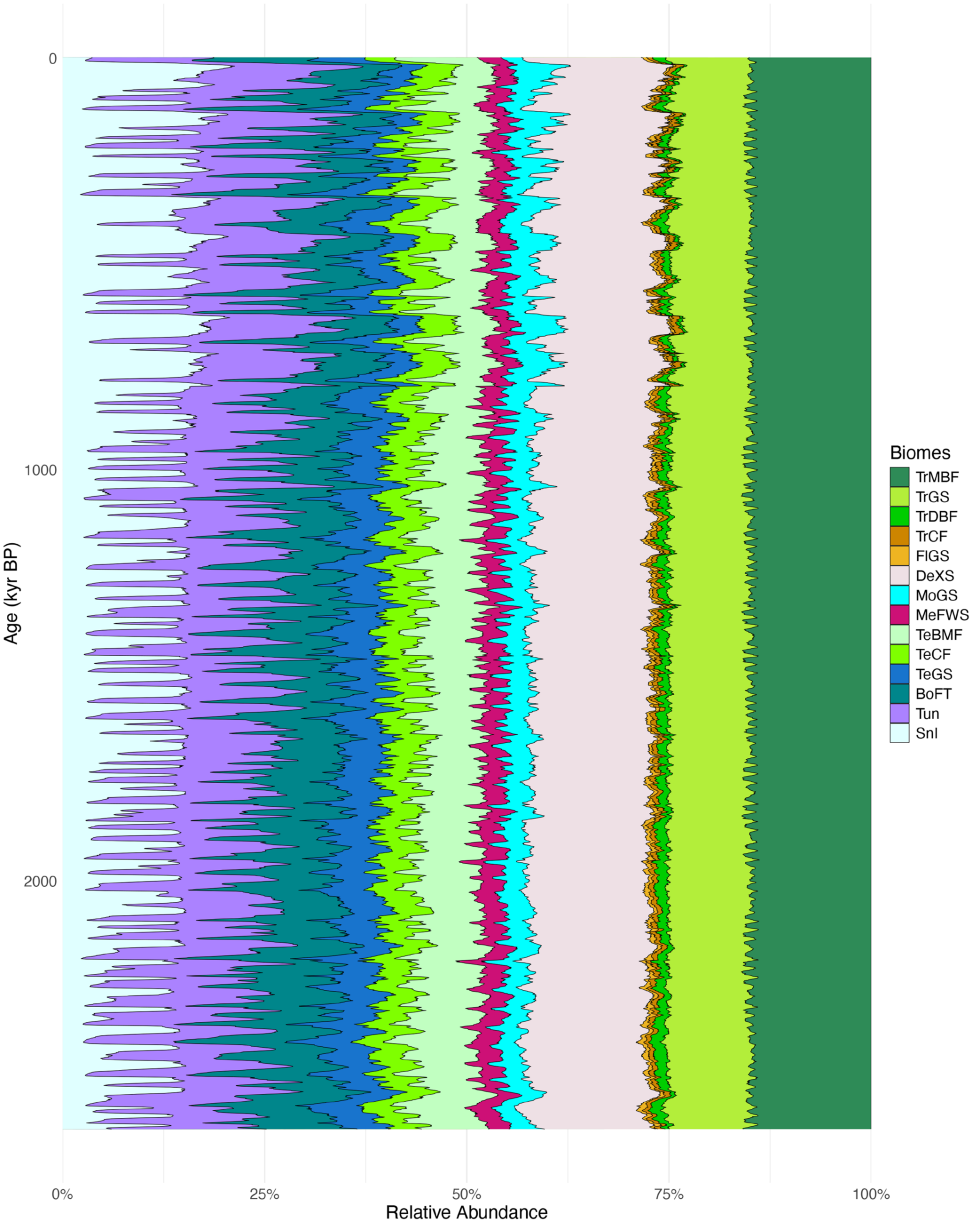
Time series of the fractional cover of different biome types. See Figure S9 for time series of each biome individually. Abbreviations of biome types are defined in Table 1.

The current biome distribution of the Olson et al. (2001) biome map was well reproduced by our biome suitability models (Figure 1, *κ* = 0.77, agreement in 79.7% of grid cells). Nonetheless, data‐model mismatches occurred, for example in the sub‐tropical and semi‐arid regions of South America and India or at the biome boundaries of the boreal forests and taiga in Russia (Figure S5). Data‐model agreement differed between biomes and was highest for deserts and xeric shrubland (*κ* = 0.85, Table S4) and lowest for flooded grassland and savanna (*κ* = 0.31, Table S4). Model evaluation using the LegacyPollen2.0 data revealed that biomes were correctly modeled for 71.8%, 72.6%, 43.6% of the pollen‐based reconstructions for 0, 6 and 21 ka (Figure S7). For BIOME6000, 57.5%, 59.8% and 36.4% were correctly classified for those periods (Figure S6). The agreement between the Olson et al. (2001) biome map and LegacyPollen2.0 or BIOME6000 for 0 ka was 70.6% and 57.1%, respectively (Figure S8). The spatial distribution of climate‐driven biome suitability, and accordingly the area covered by different biomes varied during the study period due to changes in bioclimatic conditions (Figure 1c for LGM, Figure 2, Videos S1 and S2). Regions associated with snow and ice according to the Olson et al. (2001) classification showed the largest variation ranging between 2.2% (331 ka) and 21.3% (19 ka) in interglacials and glacials (Table 1). This change represents a 89.7% decrease relative to the maximum expansion. Due to the variation of snow and ice, suitability of boreal forest/taiga and tundra also varied substantially and decreased by 73.6% and 54.3% relative to the maximum cover (15.2% and 19.7%, respectively). Tropical and subtropical moist broadleaf forest suitability showed the largest mean and median cover over the entire period (both 15.0%, Table 1) and the smallest decrease during the study period relative to the maximum cover (14.1% change). Biomes with the smallest mean cover were tropical and subtropical dry broadleaf forest, tropical and subtropical coniferous forest and flooded grassland and savanna (1.1%, 0.7% and 0.5% cover, respectively). Histograms of the cover fractions of different biomes for all time slices revealed that those distributions can be unimodal or bimodal (Figure S11). For instance, the distributions of tropical and subtropical moist broadleaf forest or tundra were unimodal whereas the distributions of boreal forest/taiga or snow and ice were bimodal.

**TABLE 1:** Ranges of coverage of different biomes. “Min”, “Median”, “Mean” and “Max” are minimum, median, mean and maximum cover during the entire study period, “Diff” is the absolute difference between minimum and maximum cover, “%Diff” is the % difference between minimum and maximum cover relative to the maximum cover, “YMin” and “YMax” are the time periods when minimum and maximum cover are reached. “Abb” is the abbreviation of the biome type used in other figures.

Biome	Abb	Min (%)	Median (%)	Mean (%)	Max (%)	Diff (%)	%Diff (ka)	YMin (ka)	YMax
(Sub)Tropical moist broadleaf forest	TrMBF	13.8	15.0	15.0	16.1	2.3	14.1	−1948	−1064
(Sub)Tropical grassland savanna	TrGS	7.7	10.0	9.9	11.6	3.9	33.9	−133	−2547
(Sub)Tropical dry broadleaf forest	TrDBF	0.3	1.1	1.1	1.8	1.5	85.4	−658	−1227
(Sub)Tropical coniferous forest	TrCF	0.5	0.7	0.7	1.1	0.7	59.8	−1170	−664
Flooded grassland and savanna	FlGS	0.2	0.5	0.5	1.4	1.2	83.6	−2174	−1072
Deserts and xeric shrubland	DeXS	12.5	14.6	14.5	15.7	3.2	20.4	−137	−2192
Montane grassland and shrubland	MoGS	1.8	3.4	3.6	7.8	6.0	76.6	−1070	−137
Mediterranean forest woodland, scrub	MeFWS	1.9	2.8	2.8	3.4	1.5	44.4	−19	−1074
Temperate broadleaf and mixed forest	TeBMF	4.3	7.1	7.4	11.1	6.7	60.7	−135	−2389
Temperate conifer forest	TeCF	3.4	4.3	4.4	6.0	2.6	42.9	−120	−2194
Temperate grassland savanna, shrubland	TeGS	2.9	5.3	5.3	9.1	6.2	68.3	−20	−1072
Boreal forest/taiga	BoFT	4.0	9.2	9.8	15.2	11.2	73.6	−228	−1447
Tundra	Tun	9.0	14.1	14.1	19.7	10.7	54.3	−2353	−1347
Snow and ice	SnI	2.2	13.0	11.0	21.3	19.1	89.7	−331	−19

### 3.2 Biome Stability

**FIGURE 3:**
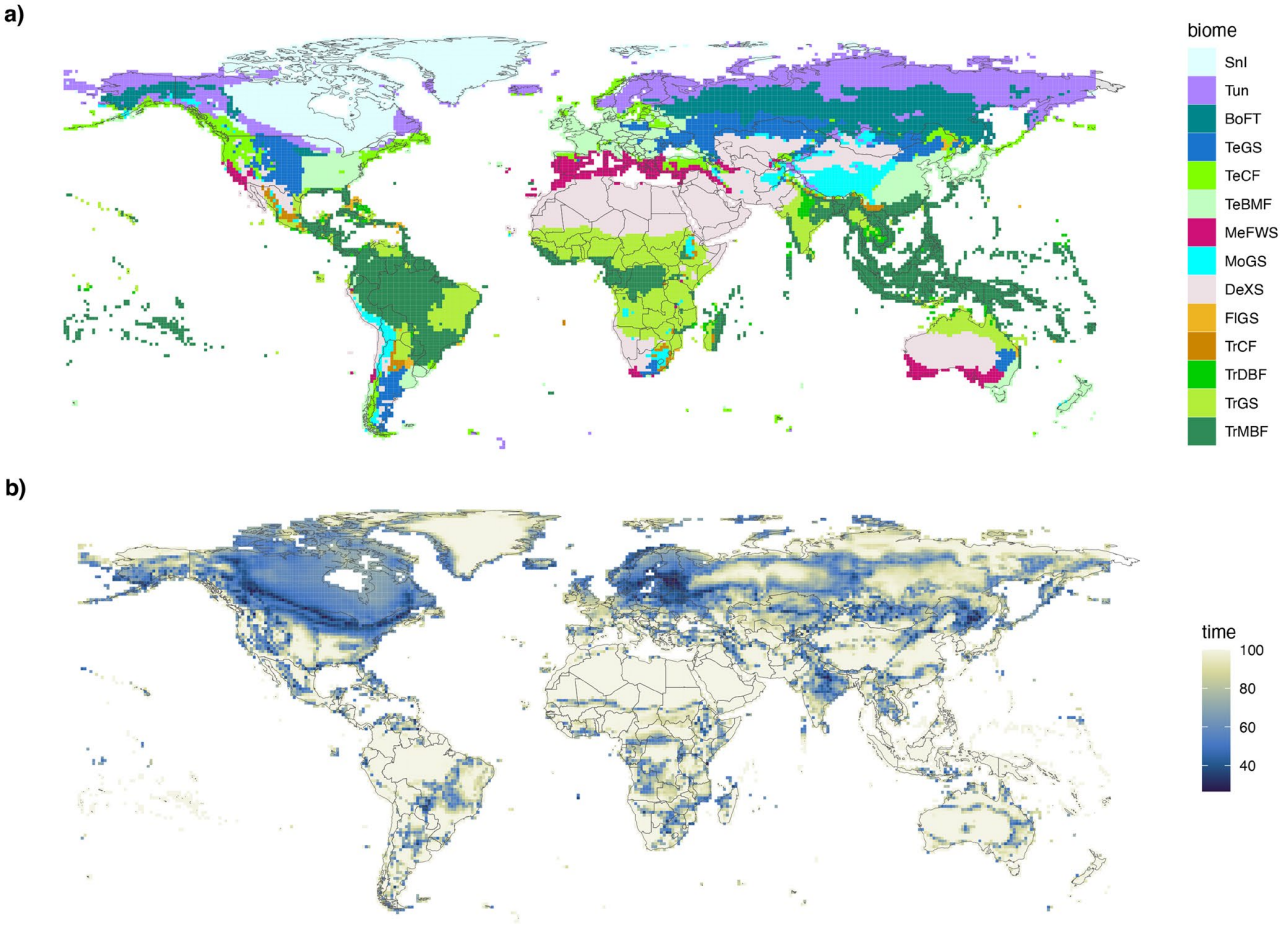
Core areas of biome changes during the Quaternary. Panel (a) shows the biome type that was modeled in most time steps during the simulation period, panel (b) shows the percent of time periods grid cells were covered by the most frequent biome type in (a). Here, 100 indicates that biomes were stable, that is, covered by the same biome type during the entire study period whereas lower values (blue) indicate core areas of biome change. Abbreviations of biome types are defined in Table 1.

Given the variation in biome suitability over time, regions of biome stability and core areas of biome change emerged (Figure 3). Core areas of biome change were identified in northern Europe and North America where periodic expansion and retreat of areas suitable for snow and ice strongly shaped biome patterns (Figure 3b, Figure S12). Yet, only 7.5% of the area ever covered by snow and ice in the study period was suitable for snow and ice during the entire study period (mostly Greenland, Table S6, Figure S12). Further core areas of biome change were modeled in tropical and subtropical regions globally where suitability for broadleaf forests decreased and suitability for grasslands and savannas increased (Figure S14). Biome stability was mostly modeled in the tropical and subtropical moist broadleaf forest around the Equator, in deserts and xeric shrubland, and in Mediterranean forest woodland and scrub (Figure S13, see Figures S15 and S16 for other biomes). Here, 47.7%, 43.8%, and 13.5% of the area ever suitable for these biomes were suitable for these biomes during the entire study period. Biome stability also occurred in boreal forests and tundra of Siberia (Figure 3, Figure S12).

The spatial patterns of biome stability changed between the first and last 150 ka of the study period. Those changes were not globally consistent, and we identified both regions where stability increased and where it decreased (Figure S10). For instance, the far north of America was more stable during the late period than during the early period. In contrast, parts of western Europe were more stable during the early period.

We compared the biome suitability modeled during the largest proportion of the time slices in the Quaternary (not necessarily longest consecutive period) and observation‐based (Olson et al. 2001) or modeled current biome types. This comparison indicated agreement of biome types in 35.5% and 23.3% of the global land surface for the observation‐based and modeled biome maps, respectively (Figure S17). For the observation‐based biome map, differences were found for instance in the boreal areas of North America and northern Europe, and, albeit to a lesser extent, in the sub‐tropics of South America, Africa and India (Figure S17a). Many of these areas in the tropics and sub‐tropics coincide with areas where the observation‐based map and the model disagree (Figure 1). For the modeled biome suitability map, the area with disagreement was smaller than for the observation‐based map, and disagreement occurred primarily in North America and northern Europe, and in smaller regions in the tropics and sub‐tropics (Figure S17b).

### 3.3 Biome Stability and Species Richness

**FIGURE 4:**
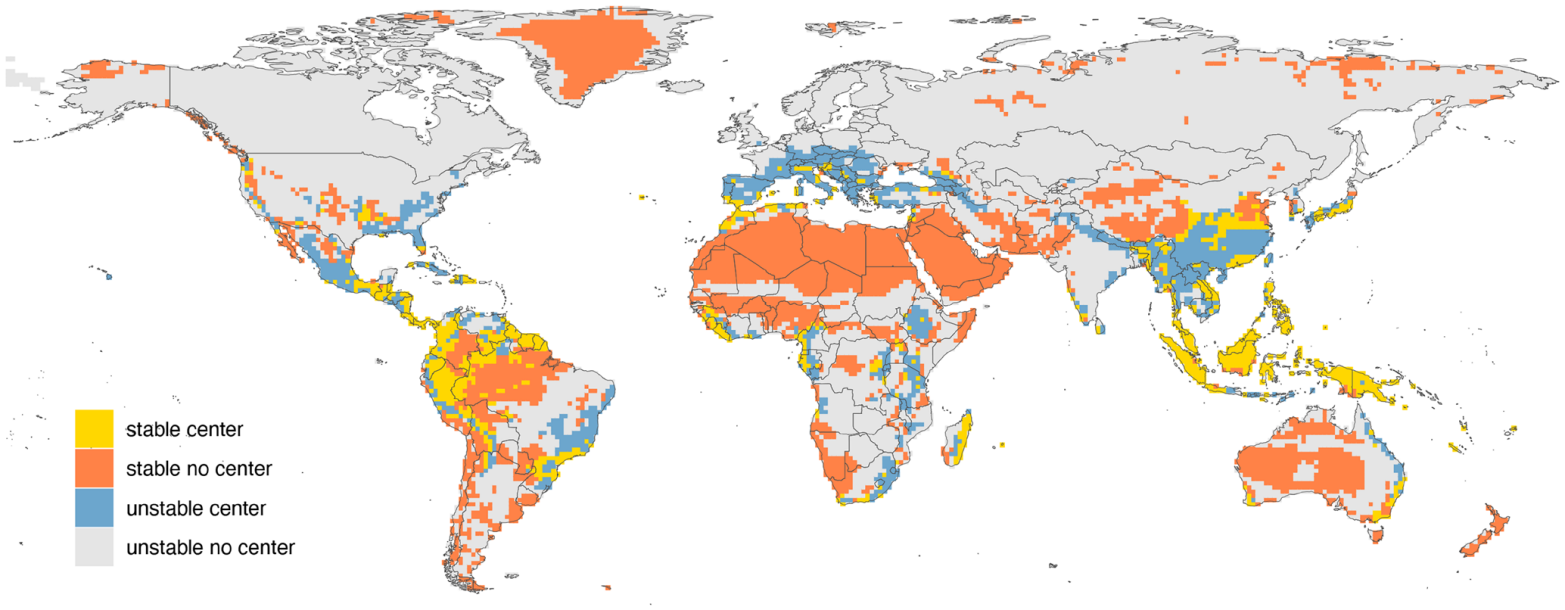
Overlap between areas with biome stability during the entire study period and centers of plant species richness according to Cai et al. (2023). In the legend, “stable” and “unstable” refer to biome stability (areas with value = 100 in Figure 3 are stable, areas with value < 100 are unstable), “center” and “no center” refer to centers of species richness. See Figure S18 for different threshold values for the definition of stability.

The relation between biome stability during the last 150 ka and current vascular plant species richness was spatially heterogeneous (Figure 4, Figure S20). The areas overlapped in the tropical rain forests of Southeast Asia, the western parts of the Amazon forests, small proportions of Mediterranean ecosystems and western China. Yet, large centers of species richness, for example in Europe, central America or southern China did not overlap with stable areas. When criteria for biome stability were released and a biome was denoted as stable when it covered a grid cell for 95% or 90% of the time, agreement between stable and species rich areas increased (Figure S18). Similarly, when reducing the number of species used to classify a grid cell as center of species richness, agreement increased, and the central Amazon rain forests were stable and centers of species richness (Figure S18). Large areas with high biome stability were associated with low diversity, particularly in the sparsely vegetated deserts of northern Africa and central Australia (Figure 4).

**FIGURE 5:**
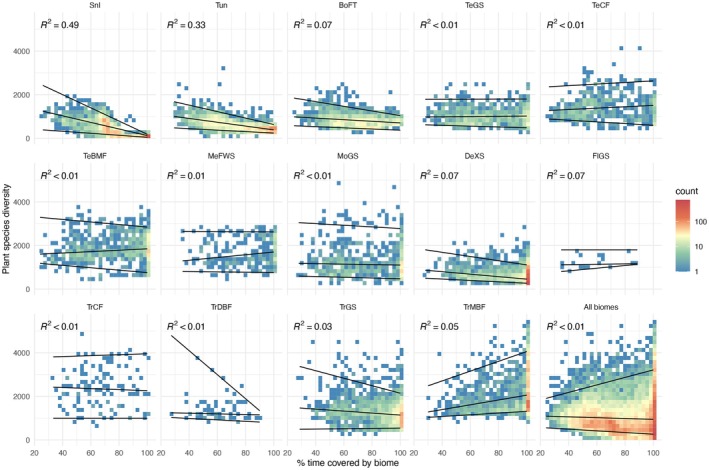
Relationship between diversity and stability per biome. Plant species richness was taken from Cai et al. (2023), % time covered by biome represents biome stability. Colors represent the number of grid cells in each bin defined by stability and species richness. Count values are on a log scale. Lines represent quantile regressions for the 5%, 50%, and 95% percentiles. Pseudo *R* ^2^ are provided for the 50% percentile regression models. All intercepts, slopes, and pseudo *R* ^2^ values are provided in Table S5. Biome types refer to current biome patterns.

The relation between species richness and stability was biome‐specific (Figures 5 and S19). Tropical and subtropical moist broadleaf forest, the biome type with the highest species richness were associated with a positive relation between richness and biome stability (Table S5). The relation was less clear for other biomes or even negative (e.g., for Deserts and xeric shrubland with low species richness). When considering all biomes, the relation was positive for the 95% quantile regression but negative for the 50% and 5% quantile regressions.

## 4 Discussion

### 4.1 Biomes of the Quaternary

We used correlative and machine learning models to generate global climate‐driven biome suitability patterns of the last 2.6 million years with a temporal resolution of 1000 years and a spatial resolution of 1°. In our approach, biome patterns are climate‐driven and follow changes in bioclimatic conditions as provided by the Barreto et al. (2023) data. Areas of biome stability were modeled in the equatorial tropical rain forests, deserts, temperate mixed forest and parts of the tundra and boreal forests. Core areas of biome change were modeled in northern Europe and Northern America where repeated expansion and retreat of areas suitable for snow and ice shaped biome patterns. Stable areas broadly agree with those identified by Huntley et al. (2021), who used the LPJ‐GUESS DGVM to simulate vegetation during the last 140 ka. Hence, two different and independent modeling approaches identified similar regions as stable and unstable. This illustrates that our approach using correlative and machine learning model for biome suitability complement previous studies. Differences occur, for example, in rainforest of South America. Our results indicate stable conditions for the entire Brazilian rainforest, while the LPJ‐GUESS simulations suggested highest stability for neighboring areas to the west and south of that.

The frequency distribution of cover fractions was uni‐modal for most biomes, indicating expansion and dieback along the borders of a stable core area of the biomes in response to climate change. In contrast, the frequency distributions of biome suitability for snow and ice, boreal forests, and temperate broad‐leaved mixed forests were bimodal. Hence, those biomes cover low or high fractions of the land surface for long periods, while intermediate cover fractions only occurred during transition phases. Specifically, during glacial periods, snow and ice replaced tundra and boreal forests, and tundra replaced temperate broad‐leaved mixed forests in Europe. Model evaluation for pollen‐based biome reconstructions from BIOME6000 (Harrison 2017) and LegacyPollen2.0 (Li et al. 2024) showed different levels of agreement (57.5% for BIOME6000 and 71.8% for LegacyPollen2.0 for current conditions). Disagreement, particularly for BIOME6000, can be explained by several reasons. First, we compared site data to modeled biome suitability in grid cells. We assumed that each grid cell is covered homogeneously by one biome type, while ignoring that the spatial heterogeneity of environmental condition, edaphic conditions, or micro‐habitats within grid cells may support a mixture of different biome types. Second, the pollen‐based reconstructions may be affected by uncertainties such as the lack of modern analogues for fossil pollen assemblages, taxonomic resolution limits, and the sensitivity of biome assignments to past CO_2_ changes (Prentice et al. 2000). Third, the climatic forcing for the LGM is more uncertain than that for the present or the mid‐Holocene, which influences the uncertainty of modeled biome suitability. Fourth, pollen‐based biome reconstructions and the Olson et al. (2001) biome map used to develop the model agreed for only 57.1% and 70.6% of the sites for BIOME6000 and LegacyPollen2.0, respectively. Those values are similar to the agreement between the pollen‐based data and our model for current conditions. As the biome matching between pollen‐based reconstructions and the Olson et al. (2001) biomes was conducted for 0 ka and then applied to other time slices, mismatches propagate to other time slices of our modeled suitability maps.

Disagreement could be reduced by using other biome maps to inform the SDMs, such as the Bonannella et al. (2023) map derived from BIOME6000 data using machine learning methods. Yet, the disadvantage of this approach is that our biome modeling would then rely on the modeled biome map by Bonannella et al. (2023) instead of the observation‐based biome map by Olson et al. (2001). Finally, biome types in pollen‐based reconstructions and the Olson et al. (2001) map disagreed, and a biome matching was required. Alternative matching approaches (Champreux et al. 2024) or an alternative biome map for model development may influence agreement. Given these uncertainties, our results should be interpreted as climate‐driven biome suitability maps rather than reconstructions. The discrepancies between model and pollen data suggest that while the models capture broad‐scale climatic shifts, they may underestimate local‐scale resilience or overemphasize biome sensitivity in regions with high environmental heterogeneity or non‐analogue conditions.

### 4.2 Biome Stability and Species Richness

It has been hypothesized that regions with low climate change velocity or biome stability can serve as refugia for species during glacial stages and explain high species diversity under current conditions (Sandel et al. 2011; Huntley et al. 2016, 2021). Our results support relations between biome stability and centers of species richness for tropical forests on the islands of Southeast Asia, the western parts of the Amazon forests and parts of Mediterranean ecosystems. These results confirm the hypothesis of these areas as evolutionary refugia, where long‐term environmental constancy was the primary driver of high taxonomic diversity because fewer species go extinct if the environmental conditions are relatively stable. Yet, global overlap of stable areas and centers of species richness was surprisingly weak, and we found large areas where stability and centers of species richness did not co‐occur. Centers of species richness across all continents, for example in southern Europe, southern China, or mainland Southeast Asia were not identified as stable areas, suggesting that stability is not universally associated with plant diversity in these regions. For example, the species richness of mainland Southeast Asia, despite biome instability, has been attributed to the “species pump” mechanism (Li and Li 2018; Dixit et al. 2023). That is, repeated Pleistocene sea‐level fluctuations and the region's complex topography may have promoted diversification in isolated populations at high sea levels and reconnection of populations at low sea levels, thereby promoting species richness, potentially in the absence of biome or climate stability. Cannon et al. (2009) argues that these fluctuations created a state of constant flux, where current rainforests represent isolated refugia that are unrepresentative of the region's expansive biogeographic past. From a vegetation perspective, this suggests that high diversity can be maintained through rapid distributional shifts and ecological tracking of suitable climates, rather than static persistence. These contrasting results illustrate that our modeling approach focusing solely on climate or biome stability can explain species richness in some regions, but not globally. A variety of paleo‐environmental, geological and topographic processes currently not included in our model should be considered in future studies.

Conclusions from analyzing overlap are biased by our definitions of stability and centers of species richness. A sensitivity analysis indicated that changes in our definitions increase the overlap between such areas. Further, we found that the 90% percentile of species richness increases with biome stability. This relation is strongly driven by tropical moist broad‐leafed forests; those forests showed the highest species richness among all biomes, and positive stability‐species richness relations for all percentiles. This finding agrees with Huntley et al. (2021), showing that biome stability is a strong predictor for vertebrate diversity in regions with high diversity, even though patterns of biome stability did not fully agree in both studies. Overlap also depends on the species richness map used for comparisons. The Cai et al. (2023) map represents species richness at the regional scale, which is more comparable to the 1° resolution of our study. Regions such as the central Amazon forests are not identified as diversity hotspots in the Cai et al. (2023) map, in contrast to the neighboring northern Andes. This discrepancy emphasizes the role of topographic complexity for species richness. While the Amazonian lowlands are more homogeneous with low environmental heterogeneity, mountain areas such as the Andes harbor large environmental variability on small areas and provide a range of vertical niches. High species richness in such topographically complex regions can be maintained because plants can track their climatic envelopes during periods of climate change through short‐distance altitudinal migrations rather than long‐distance latitudinal shifts (Rahbek et al. 2019; Stein et al. 2014). Hence, plants can persist in locally stable biomes or climates. Our results suggest that biome stability can be a key driver for species persistence in lowland refugia; species richness in mountain systems can be driven by niche diversity and topographic buffering against Quaternary climate oscillations.

Sabatini et al. (2022) provide species richness at the plot level and show high species richness in the Amazon forest that would therefore be classified as stable centers of species richness in our analysis (Figures S21 and S22). While plot‐level data provide high‐precision local species richness information, we prioritized the Cai et al. (2023) map to minimize the spatial scale mismatch between species richness data and the 1‐degree resolution of our bioclimatic predictors, thereby reducing potential noise from local‐scale environmental heterogeneity. The Cai et al. (2023) map is based on machine learning and does not capture the full species richness at plot level.

Mismatches between stability and species richness hotspots may also arise when total diversity does not align with the diversity of specialists or endemics, or diversity of different functional groups such as forest or non‐forest. The climate or habitat stability hypothesis is expected to be most relevant for species that depend heavily on specific environmental conditions, such as specialists and endemics (Sandel et al. 2011). In contrast, overall native species richness also includes many generalist species, which likely weakens the observed relationship. Generalists may persist under a wide range of bioclimatic conditions while specialists and endemics are present or absent. A high proportion of generalists may therefore attenuate the statistical signal of specialists and endemics.

Sandel et al. (2011) showed that areas with low climate change velocity since the last glacial maximum were refuges for many small‐ranged animal species while areas with high climate change velocity lack endemic animal species with small ranges. Our core areas of biome change do not fully agree with those areas of high climate change velocity. Sandel et al. (2011) identified mainly North America and Scandinavia as areas with high climate change velocity, while we also identified areas in the tropics and subtropics as core areas of biome change.

Patterns of species richness have been studied in the context of climate velocity or climate stability, and in the context of biome stability. Huntley et al. (2016) and Colville et al. (2020) found a stronger relation between biome stability and diversity than with climate stability, and we therefore followed that approach. This stronger relation has been associated with multiple processes. Lagged responses of vegetation dynamics imply that vegetation and biomes will continue to change even if climate stabilized (Bertrand et al. 2016; Scheiter et al. 2020), such that biome stability does not reflect climate stability. However, we assume that biome stability in our model is primarily driven by bioclimatic suitability, acknowledging that this approach equates stable climate with stable biomes and may not fully capture lagged vegetation responses. Further, species richness or disturbances such as fire may buffer the response of vegetation to climate change and maintain it in a disequilibrium or alternative vegetation state (Pausas and Bond 2020) under the given climate conditions. It has been shown that in different biomes, areas with higher plant diversity are less sensitive to temperature variability (Oliveira et al. 2022), potentially decoupling biome states from climate. We acknowledge that in our model, such a decoupling is not possible as biome suitability is based on bioclimate. On the other hand, factors such as invasive species, changes in disturbance regimes, or, under current and historic conditions, due to human activities and land use may destabilize biomes and enhance their susceptibility to biome changes despite stable climate.

### 4.3 Drivers of Paleo‐Biome Distributions

Our modeling approach is only based on the current distribution of biomes (Olson et al. 2001) and on bioclimatic variables derived from precipitation and temperature. Yet, other biotic and abiotic drivers, including atmospheric CO_2_, solar radiation, fire, and animals, have been shown to influence vegetation. Ignoring these drivers can explain mismatches between reconstructed and modeled biome patterns, for example, in the South East United States or in North West India. Including more bioclimatic variables in our modeling approach may also improve data‐model agreement, but we aimed for a parsimonious model with a reduced number of bioclimatic predictors.

During the study period, CO_2_ was between approximately 180 and 300 ppm (Berends et al. 2021) while it is currently at approximately 420 ppm. CO_2_ has strong impacts on plant growth, particularly of C_3_ plants (Ehleringer et al. 1997). Low CO_2_ during glacial periods may have further reduced the expansion of forests (Woillez et al. 2011; Harrison and Prentice 2003), compared to our results. To capture the effects of CO_2_, process‐based dynamic vegetation models have been used (Harrison and Prentice 2003; Allen et al. 2020; Huntley et al. 2023; Woillez et al. 2011; Zeller et al. 2023).

Fire is an important component of ecosystems, for example in tropical savannas (Bond and Keeley 2005). While information on fire, such as burnt area (van der Werf et al. 2017) can be included to create biome models for current conditions, information on past or future fire activity at a global scale and at high temporal resolution is missing. Therefore, fire was not considered in our analysis. Yet, fire has been included in studies using process‐based models. For example, Scheiter et al. (2012) highlighted the importance of fire for C_4_ expansion during the late Miocene, and Sato et al. (2021) found that fire and low CO_2_ may have caused the dieback of Amazon rain forests and expansion of savannas and grasslands into the Amazon rain forests during the LGM. This contrasts with our results where the Amazon rain forest was stable during the Quaternary, and savanna corridors did not occur. However, our results are consistent with the results from the process‐based LPJ‐GUESS DGVM that included plant‐physiological effects, the atmospheric CO_2_ concentration, and fire (Huntley et al. 2021, 2023). These results did not show savanna corridors or widespread fragmentation of the tropical forest over the last 140 and 800 ka years, respectively.

Our results indicate that the Sahara desert was stable during the entire study period while reconstructions indicate Sahara greening and C_3_ vegetation during three periods in the last 192 ka (Castaneda et al. 2009). In addition to the African monsoon, such greening events have been explained by climate‐vegetation feedbacks (Claussen and Gayler 1997). Such feedbacks cannot be represented by our modeling approach or stand‐alone DGVMs (Huntley et al. 2023) but with fully coupled biosphere‐atmosphere models. A further reason for mismatches between modeled and reconstructed biome patterns may be uncertainties in the Barreto et al. (2023) climate reconstructions that we used for the modeling. If those data do not represent more humid conditions in the Sahel during respective periods, our model does not produce a green Sahara.

### 4.4 Modeling Suitability for Snow and Ice

In our approach, we treated ice sheets as a biome and modeled their climate‐driven suitability and extent similar to biomes representing vegetation. We argue that this approach is plausible. Ice sheets require low temperatures and, on land, sufficient precipitation to build up and persist, and therefore have a well‐defined climatic niche. According to our model, ice sheets occur below −3.8°C and at median and maximum MAP of 125 and 1718 mm, respectively. Nonetheless, this approach does not reflect the full complexity of the expansion and retreat of ice sheets. Mechanistic models are available to model ice sheet dynamics (e.g., Winkelmann et al. 2011). Yet, this limitation is also true for biomes representing vegetation. Both our correlative and machine learning biome models and DGVMs are simplifications of real vegetation dynamics and do not perfectly represent niches and biogeographic patterns of biomes.

Despite these simplifications, the model reproduced repeated expansions and retreats of areas suitable for snow and ice in northern latitudes in response to climate. Yet, previous reconstructions and models showed that ice sheets expanded further to the South than in our model (e.g., Gowan et al. 2021). Further, glaciers, for example in the Alps, were not modeled. The Olson et al. (2001) biome map underlying our model does not represent those glaciers, such that the niche is not represented by the model. This caveat can be resolved by creating models with high resolution biome maps that include glaciers and by using high resolution climate data.

### 4.5 Limitations of Modeling Approach

Using correlative and machine learning approaches to model biome suitability implies several caveats. Biome reconstructions based on those approaches are fully climate‐driven, in contrast to process‐based DGVMs (Huntley et al. 2023; Zeller et al. 2023). Therefore, our approach is flexible, computationally less demanding and can be applied to any biome map, time period or spatial resolution, given that appropriate climate data are available. It also includes a representation of the potential climatic niche of snow and ice, that have to be provided by external data in DGVM studies. Therefore, our approach complements previous DGVM studies. Ideally, the strengths of process‐based and correlative models are combined in future work, for example by comparisons of modeled biome patterns, or by using DGVM results as additional layers in correlative models.

Ecosystems are typically not in equilibrium with the environment but lag behind climate change (Loarie et al. 2009; Scheiter et al. 2020). Time lags have been proposed to be an important driver of Quaternary biome distributions in particular in areas that are heavily affected by strong and fast climatic changes associated with Heinrich and Dansgaard‐Oeschger events, such as western Europe (Huntley et al. 2013). Under changing climate, species need to migrate to follow shifts in their environmental niche. Plant species migration is constrained by seed dispersal and biotic interactions such as competition. Such processes have been considered in process‐based ecosystem models (Sato and Ise 2012; Lehsten et al. 2014) while in correlative and machine learning approaches, migration has been represented by assuming general dispersal distances applying to all species (e.g., Hof et al. 2018). The Hof et al. (2018) approach does not reflect the large differences in reconstructed migration rates of different tree species.

Applying correlative and machine learning approaches at paleo‐ecological time scales implies the assumption that changes within the biomes due to species re‐assembly or evolution can be neglected. While such processes are relevant during the 2.6 Ma time period considered in this study (e.g., Levsen et al. 2012; Tomasello et al. 2020; Kadereit and Abbott 2021), we argue that biomes represent vegetation at an aggregated level where replacement or evolution of single species may not necessarily influence the biome type. Niche conservatism ensures that traits of novel species are similar to traits of ancestral species such that the biome structure and functioning are conserved (Crisp et al. 2009). Our approach implies that the niches of biomes are conserved between interglacial climate used to create the model and glacial climate, and potential adaptations of the niches were not considered. Yet, it has been shown that past and future climate change causes regions with no‐analogue climates, and the performance of correlative and machine learning approaches may be lower in such regions, implying misleading results (Williams and Jackson 2007; Veloz et al. 2012). The reliability of our biome suitability estimates must be interpreted with caution for periods or regions characterized by non‐analogue climates. Since our models were calibrated on contemporary climate‐vegetation relationships, the projection into deep‐time involves inherent extrapolation risks. Therefore, the predicted suitability maps may reflect a more conservative estimate of potential biome distributions. Niche conservatism and no‐analogue climates also affect reconstructions derived by DGVM. While those models are process‐based, they are also parameterized using observation‐based data for current climate conditions, and it is assumed those parameters are also valid for past conditions.

The coarse 1° spatial resolution of our analysis may explain mismatches between pollen‐based reconstructions and model results. For instance, a grid cell classified as forest may also include patches with diverse non‐forest vegetation. This may falsify the stability‐diversity relationship. Further, grid cells classified as unstable may still contain stable refugia that ensure species persistence. The resolution was prescribed by the Barreto et al. (2023) bioclimatic data. Climate data with higher spatial resolution is, to our knowledge, not available for the entire Quaternary. Yet, our approach could be applied for specific regions or time periods, given that high‐resolution data is available.

## 5 Conclusions

We created a time series of climate‐driven biome suitability maps for the last 2.6 million years at 1000 year temporal resolution using correlative and machine learning approaches, to gain insights into the dynamics and stability of terrestrial biomes during this period. Correlative and machine learning approaches can reproduce current biome patterns with high accuracy; they include the dynamics of areas suitable to support snow and ice, they can be informed by any available bioclimatic and biome data set, and the high performance allows modeling biomes at high spatial and temporal resolution. These features imply that correlative and machine learning models are a flexible and objective approach to complement biome reconstructions from process‐based DGVMs. Despite mismatches with reconstructions—a common caveat of all modeling approaches—our biome suitability maps and our modeling approach are valuable for future research. For example, modeled paleo‐biomes have been used to study hominin expansion (Zeller et al. 2023), and our maps can be used to revisit those results. Further, they can contribute to the long‐standing question whether climate change, and accordingly biome change, or human impacts drove large‐scale Quaternary extinctions of the megafauna (Sandom et al. 2014; Lemoine et al. 2023). Our modeling approach can also be applied to study micro‐refugia at high spatial resolution if suitable environmental data are available, or to model future biome patterns and identify areas that are susceptible to biome shifts and species richness loss. Taken together, uncertainties arising from model transferability, simplification of biome definitions, and scale mismatches suggest that our results are most robust in identifying broad spatial patterns rather than precise biome boundaries or transitions. Overall, we found weak support for the hypothesis that biome stability during the Quaternary explains high plant species richness in current centers of species richness globally. We conclude that further research is required to understand current species richness patterns and to inform the development of strategies to protect species richness under future climate change.

## Author Contributions

**Simon Scheiter:** conceptualization (lead), data curation (lead), formal analysis (lead), investigation (lead), methodology (lead), project administration (lead), resources (lead), software (lead), supervision (lead), validation (lead), visualization (lead), writing – original draft (lead), writing – review and editing (equal). **Julia Brugger:** conceptualization (supporting), writing – review and editing (equal). **Thomas Hickler:** conceptualization (supporting), writing – review and editing (equal).

## Funding

The authors have nothing to report.

## Conflicts of Interest

The authors declare no conflicts of interest.

## Supporting information


**Figure S1:** Drill core sites from Hooghiemstra et al. (2022) used to extract time series of modeled biome types.
**Figure S2:** Biome types at drill core sites from Hooghiemstra et al. (2022), part 1.
**Figure S3:** Biome types at drill core sites from Hooghiemstra et al. (2022), part 2.
**Figure S4:** Biome types at drill core sites from Hooghiemstra et al. (2022), part 3.
**Figure S5:** Agreement of observation‐based and modeled biome distributions.
**Figure S6:** Comparison of modeled biomes and the BIOME6000 data.
**Figure S7:** Comparison of modeled biomes and the LegacyPollen2.0 data.
**Figure S8:** Comparison of the Olson et al. (2001) biomes and pollen‐derived biomes for 0 ka.
**Figure S9:** Time series of the fractional cover of different biome types.
**Figure S10:** Hotspots of biome change during the last and first 150 ka of the study period.
**Figure S11:** Histograms of biome cover for entire study period.
**Figure S12:** Percent time covered by biome during entire study period.
**Figure S13:** Percent time covered by biome during entire study period.
**Figure S14:** Percent time covered by biome during entire study period.
**Figure S15:** Percent time covered by biome during entire study period.
**Figure S16:** Percent time covered by biome during entire study period.
**Figure S17:** Agreement between the current biome distribution and the most frequent biome distribution for the entire study period.
**Figure S18:** Overlap between areas with biome stability.
**Figure S19:** Biome‐specific species richness.
**Figure S21:** Overlap between areas with biome stability during the entire study period and centers of plant species richness according to Sabatini et al. (2021).
**Figure S22:** Relationship between diversity and stability per biome.
**Table S1:** Bioclimatic variables available for the study.
**Table S2:** Matching between Olson et al. (2001) biomes and BIOME6000 biomes (Harrison 2017).
**Table S3:** Matching between Olson et al. (2001) biomes and LegacyPollen2.0 biomes (Li et al. 2024).
**Table S4:** Data‐model agreement per biome for current conditions.
**Table S5:** Quantile regression for diversity and stability (Figure 5 in main text).
**Table S6:** Biome stability during entire study period.

**Data S1:** Biome types for drill core sites, sites taken from Hooghiemstra et al. (2022) Scientific drilling, Table S1.

**Video S1:** Animation of modeled biome suitability from past to present (2.6 Ma ago to year 1950) at 1000 year temporal resolution.

**Video S2:** Animation of modeled biome suitability from present to past (year 1950 to 2.6 Ma ago) at 1000 year temporal resolution.

## Data Availability

The Olson et al. (2001) biome map was taken from Fischer et al. (2022): https://doi.org/10.5061/dryad.hqbzkh1jm. The bioclimatic variables were taken from Barreto et al. (2023): https://figshare.com/s/d45714f7212de7225fe2. The BIOME6000 biome reconstructions were taken from Harrison (2017): https://researchdata.reading.ac.uk/99/. The LegacyPollen2.0 biome reconstructions were taken from Li et al. (2005): https://doi.org/10.1594/PANGAEA.965907. The diversity data were taken from Cai et al. (2023): https://gift.unigoettingen.de/shiny/predictions/. All R scripts used for the SDM, data analysis and creating figures were uploaded to Zenodo: Scheiter, Brugger, and Hickler (2025): https://zenodo.org/records/14801838.
