# Supplementary material for: The Effects of Biome Stability During the Quaternary on Plant Diversity
Source: Ecol Evol. 2026 Jun 30;16(7):e73884. doi: 10.1002/ece3.73884 (PMC13316459; doi:10.1002/ece3.73884)
Supplement: Supplementary file 1 — Figure S1: Drill core sites from Hooghiemstra et al. (2022) used to extract time series of modeled biome types. Figure S2: Biome types at drill core sites from Hooghiemstra et al. (2022), part 1. Figure S3: Biome types at drill core sites from Hooghiemstra et al. (2022), part 2. Figure S4: Biome types at drill core sites from Hooghiemstra et al. (2022), part 3. Figure S5: Agreement of observation‐based and modeled biome distributions. Figure S6: Comparison of modeled biomes and the BIOME6000 data. Figure S7: Comparison of modeled biomes and the LegacyPollen2.0 data. Figure S8: Comparison of the Olson et al. (2001) biomes and pollen‐derived biomes for 0 ka. Figure S9: Time series of the fractional cover of different biome types. Figure S10: Hotspots of biome change during the last and first 150 ka of the study period. Figure S11: Histograms of biome cover for entire study period. Figure S12: Percent time covered by biome during entire study period. Figure S13: Percent time covered by biome during entire study period. Figure S14: Percent time covered by biome during entire study period. Figure S15: Percent time covered by biome during entire study period. Figure S16: Percent time covered by biome during entire study period. Figure S17: Agreement between the current biome distribution and the most frequent biome distribution for the entire study period. Figure S18: Overlap between areas with biome stability. Figure S19: Biome‐specific species richness. Figure S21: Overlap between areas with biome stability during the entire study period and centers of plant species richness according to Sabatini et al. (2021). Figure S22: Relationship between diversity and stability per biome. Table S1: Bioclimatic variables available for the study. Table S2: Matching between Olson et al. (2001) biomes and BIOME6000 biomes (Harrison 2017). Table S3: Matching between Olson et al. (2001) biomes and LegacyPollen2.0 biomes (Li et al. 2024). Table S4: Data‐model agreement per biome for curr [file ECE3-16-e73884-s001.pdf]

The effects of biome stability during the Quaternary on plant diversity - Supplementary materials -

Simon Scheiter, Julia Brugger, Thomas Hickler

16th June 2026

Table S1: Bioclimatic variables available for the study. Data were created by Barreto et al. (2023). BIO2 (Mean Diurnal Temperature Range) and BIO3 (Isothermality (BIO2/BIO7)) were not available. Bioclimatic data are available for the last 5 million years at a 1000 year temporal and 1° spatial resolution.

BIO1	Annual Mean Temperature
BIO4	Temperature Seasonality (standard deviation x100)
BIO5	Max Temperature of Warmest Month
BIO6	Min Temperature of Coldest Month
BIO7	Temperature Annual Range (BIO5-BIO6)
BIO8	Mean Temperature of Wettest Quarter
BIO9	Mean Temperature of Driest Quarter
BIO10	Mean Temperature of Warmest Quarter
BIO11	Mean Temperature of Coldest Quarter
BIO12	Annual Precipitation
BIO13	Precipitation of Wettest Month
BIO14	Precipitation of Driest Month
BIO15	Precipitation Seasonality (Coefficient of Variation)
BIO16	Precipitation of Wettest Quarter
BIO17	Precipitation of Driest Quarter
BIO18	Precipitation of Warmest Quarter
BIO19	Precipitation of Coldest Quarter

Table S2: Matching between Olson et al. (2001) biomes and BIOME6000 biomes (Harrison 2017). The matching was obtained by maximizing the agreement between site data in BIOME6000 and the modeled biome type in the grid cell of the BIOME6000 sites for 0 ka.

BIOME6000 biome types	Olson et al. (2001) biome types
cold deciduous forest	Boreal forest/taiga
cold evergreen needleleaf forest	Boreal forest/taiga
cold mixed forest	Temperate broadleaf and mixed forest
cool evergreen needleleaf forest	Temperate conifer forest
cool grassland	Montane grassland and shrubland
cool mixed forest	Temperate broadleaf and mixed forest
cool-temperate evergreen needleleaf and mixed forest	Temperate conifer forest
cool-temperate rainforest	Temperate broadleaf and mixed forest
desert	Deserts and xeric shrubland
erect dwarf shrub tundra	Tundra
graminoid and forb tundra	Tundra
low and high shrub tundra	Tundra
prostrate dwarf shrub tundra	Tundra
steppe	Temperate grassland savanna and shrubland
temperate deciduous broadleaf forest	Temperate broadleaf and mixed forest
temperate evergreen needleleaf forest	Temperate broadleaf and mixed forest
temperate evergreen needleleaf open woodland	Temperate grassland savanna and shrubland
temperate grassland and xerophytic shrubland	Tropical and subtropical moist broadleaf forest
temperate or tropical grassland and xerophytic shrubland	Temperate broadleaf and mixed forest
temperate sclerophyll woodland and shrubland	Temperate broadleaf and mixed forest
temperate xerophytic woods/scrub	Tropical and subtropical grassland savanna and shrubland
tropical deciduous broadleaf forest and woodland	Tropical and subtropical moist broadleaf forest
tropical evergreen broadleaf forest	Tropical and subtropical moist broadleaf forest
tropical savanna	Tropical and subtropical grassland savanna and shrubland
tropical semi-evergreen broadleaf forest	Tropical and subtropical moist broadleaf forest
tundra	Montane grassland and shrubland
warm-temperate evergreen broadleaf and mixed forest	Mediterranean forest woodland and scrub
warm-temperate evergreen broadleaf forest	Tropical and subtropical moist broadleaf forest
warm-temperate rainforest	Tropical and subtropical moist broadleaf forest
wet sclerophyll forest	Temperate broadleaf and mixed forest
xerophytic woods/scrub	Mediterranean forest woodland and scrub
cushion forb tundra	Snow and ice

Table S3: Matching between Olson et al. (2001) biomes and LegacyPollen2.0 biomes (Li et al. 2024). The matching was obtained by maximizing the agreement between site data in LegacyPollen2.0 and the modeled biome type in the grid cell of the LegacyPollen2.0 sites for 0 ka.

LegacyPollen2.0 biome types	Olson et al. (2001) biome types
Tropical forest (TRFO)	Tropical and subtropical moist broadleaf forest
(Warm) savanna and dry woodland (SAVA)	Tropical and subtropical grassland savanna and shrubland
Tropical forest (TRFO)	Tropical and subtropical dry broadleaf forest
Temperate forest (TEFO)	Tropical and subtropical coniferous forest
Temperate forest (TEFO)	Flooded grassland and savanna
Grassland and dry shrubland (STEP)	Deserts and xeric shrubland
Grassland and dry shrubland (STEP)	Montane grassland and shrubland
Temperate forest (TEFO)	Mediterranean forest woodland and scrub
Temperate forest (TEFO)	Temperate broadleaf and mixed forest
Temperate forest (TEFO)	Temperate conifer forest
Temperate forest (TEFO)	Temperate grassland savanna and shrubland
Boreal forest (BOFO)	Boreal forest/taiga
Boreal forest (BOFO)	Tundra
Tundra and polar desert (TUND)	Snow and ice

Table S4: Data-model agreement per biome for current conditions. Agreement was quantified using the κ statistics.

biome	κ
Tropical and subtropical moist broadleaf forest	0.81
Tropical and subtropical grassland savanna and shrubland	0.75
Montane grassland and shrubland	0.71
Tropical and subtropical dry broadleaf forest	0.51
Tropical and subtropical coniferous forest	0.36
Flooded grassland and savanna	0.31
Temperate broadleaf and mixed forest	0.77
Mediterranean forest woodland and scrub	0.70
Temperate conifer forest	0.48
Temperate grassland savanna and shrubland	0.74
Deserts and xeric shrubland	0.85
Boreal forest/taiga	0.82
Tundra	0.81
Snow and ice	0.84

Table S5: Quantile regression for diversity and stability (Fig. 5 in main text). I_p, S_p and R_p are intercept, slope and pseudo R^2 values for the p quantile regression.

biome	I_{10}	S_{10}	R_{10}	I_{50}	S_{50}	R_{50}	I_{90}	S_{90}	R_{90}
SnI	719	-6.9	0.09	1631	-15.08	0.4	2793	-26.28	0.41
Tun	700	-4.34	0.05	1260	-8.77	0.22	1921	-13.61	0.3
BoFT	764	-3.93	0.02	1072	-3.67	0.02	1897	-9.48	0.1
TeGS	702	-1.19	0.01	960	0.58	0	1630	-0.84	0
TeCF	1028	-3.18	0.03	1195	3.11	0.01	2044	2.86	0
TeBMF	1248	-1.64	0	1528	3.19	0.01	3326	-7.29	0
MeFWS	952	1.04	0	1072	6.31	0.02	2638	-1.1	0
MoGS	696	-1.33	0.01	1209	-1.06	0	2286	-0.46	0
DeXS	709	-4.28	0.06	1025	-5.68	0.05	1933	-10.16	0.04
FlGS	593	6.1	0.19	1083	0.92	0.01	1091	8.5	0.06
TrCF	1435	-4.7	0.01	2500	-2.39	0	2647	15.51	0.04
TrDBF	1285	-5.5	0.03	1285	-1.48	0	2020	2.13	0.01
TrGS	715	-0.7	0	1596	-4.54	0.02	3082	-12.99	0.04
TrMBF	930	4.62	0.02	952	11.03	0.02	1182	25.66	0.04
All biomes	843	-5.39	0.02	1136	-1.91	0	1305	12.67	0.02

Table S6: Biome stability during entire study period. For each grid cell, the percent of time slices in which it is covered by the target biome was calculated. Grid cells that were never covered by the target biome were removed. Then, the percent of grid cell was calculated in which a biome was represented in 100% (p100), 99% (p99), 98% (p98), 95% (p95) or 90% (p90) of the time periods. High values indicate that a large proportion of the biome is stable during the entire study period, whereas small values indicate that a biome undergoes frequent biome changes during the study period.

biome	p100	p99	p98	p95	p90
Tropical and subtropical moist broadleaf forest	47.7	52.8	54.2	56.9	59.3
Tropical and subtropical grassland savanna and shrubland	9.4	16.7	20.0	26.0	33.2
Tropical and subtropical dry broadleaf forest	0.0	0.0	0.0	0.1	0.2
Tropical and subtropical coniferous forest	0.1	0.7	0.7	1.1	2.1
Flooded grassland and savanna	0.0	0.0	0.0	0.0	0.1
Deserts and xeric shrubland	43.8	50.2	52.1	54.9	58.3
Montane grassland and shrubland	4.6	6.3	6.6	7.1	8.4
Mediterranean forest woodland and scrub	13.5	18.2	19.8	22.3	25.7
Temperate broadleaf and mixed forest	9.0	12.7	14.1	16.7	19.8
Temperate conifer forest	1.6	2.7	3.1	4.0	5.0
Temperate grassland savanna and shrubland	2.1	4.2	5.1	6.4	8.7
Boreal forest/taiga	0.0	0.8	1.3	4.1	8.4
Tundra	1.5	5.4	7.2	9.7	12.8
Snow and ice	7.5	9.7	10.4	11.9	14.1

References

Barreto E, Holden PB, Edwards NR, Rangel TF (2023) PALEO-PGEM-Series: A spatial time series of the global climate over the last 5 million years (Plio-Pleistocene). Global Ecology and Biogeography, **32**, 1034–1045.

Cai L, Kreft H, Taylor A, et al. (2023) Global models and predictions of plant diversity based on advanced machine learning techniques. New Phytologist, **237**, 1432–1445.

Harrison S (2017) BIOME 6000 DB classified plotfile version 1. University of Reading. Dataset.

Hooghiemstra H, Pérez GS, Torres Torres V, Berrío JC, Lourens L, Flantua SGA (2022) 60 years of scientific deep drilling in Colombia: the north Andean guide to the Quaternary. Scientific Drilling, **30**, 1–15.

Li C, Dallmeyer A, Ni J, et al. (2024) Global biome changes over the last 21,000 years inferred from model-data comparisons. EGUsphere, **2024**, 1–26.

Olson DM, Dinerstein E, Wikramanayake ED, et al. (2001) Terrestrial ecoregions of the world: A new map of life on earth: A new global map of terrestrial ecoregions provides an innovative tool for conserving biodiversity. BioScience, **51**, 933–938.

Sabatini FM, Lenoir J, Hattab T, et al. (2021) sPlotOpen - An environmentally balanced, open-access, global dataset of vegetation plots. Global Ecology and Biogeography, **30**, 1740–1764.

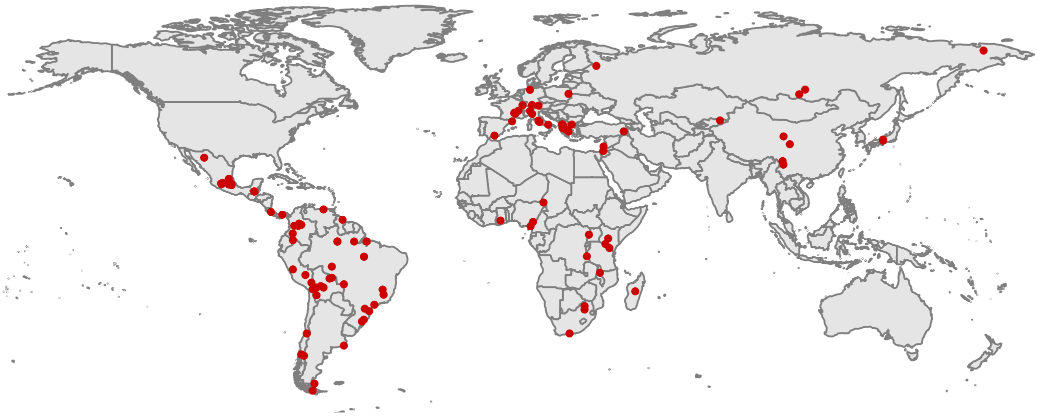

Figure S1: Drill core sites from Hooghiemstra et al. (2022) used to extract time series of modeled biome types.

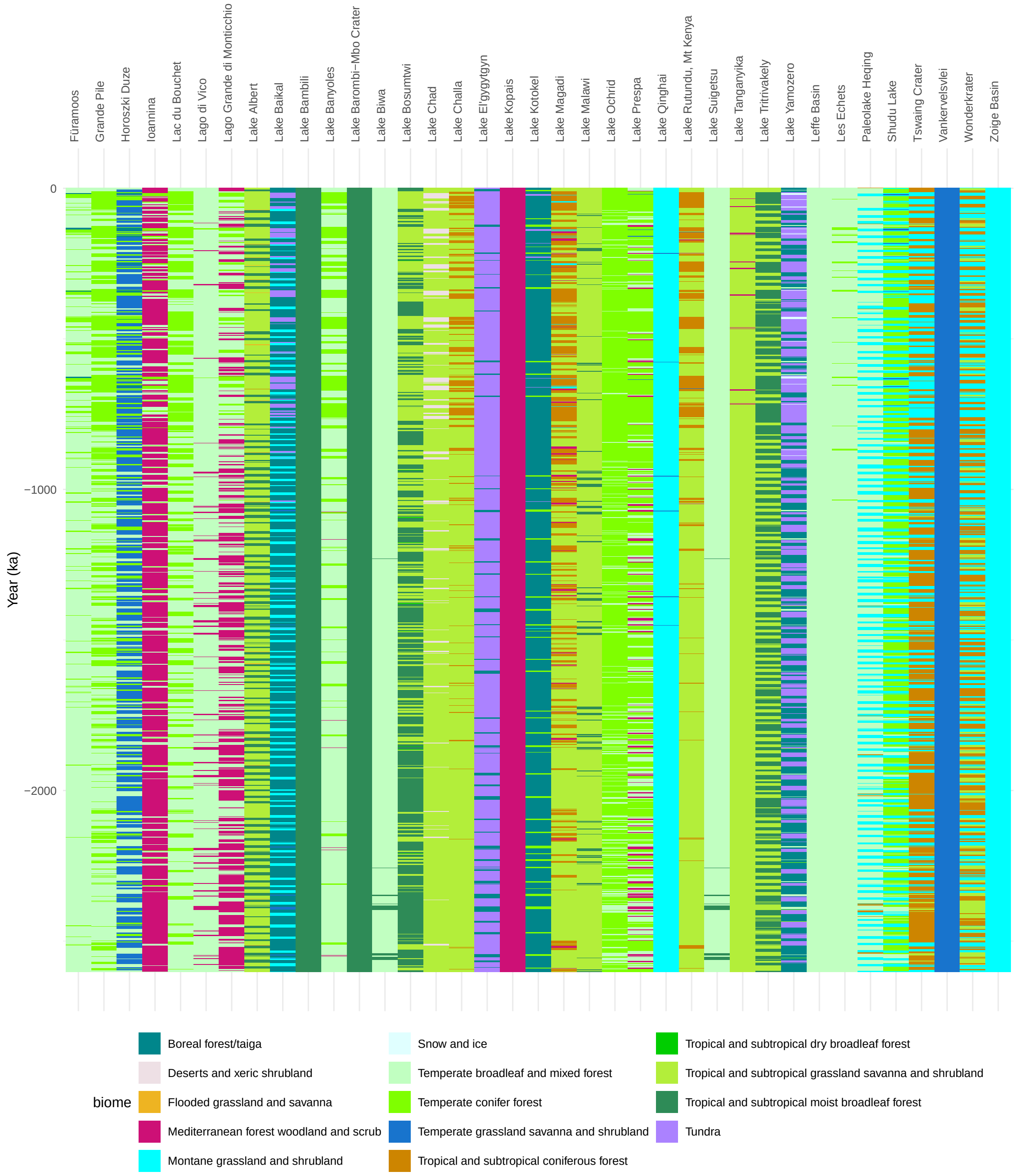

Figure S2: Biome types at drill core sites from Hooghiemstra et al. (2022), part 1.

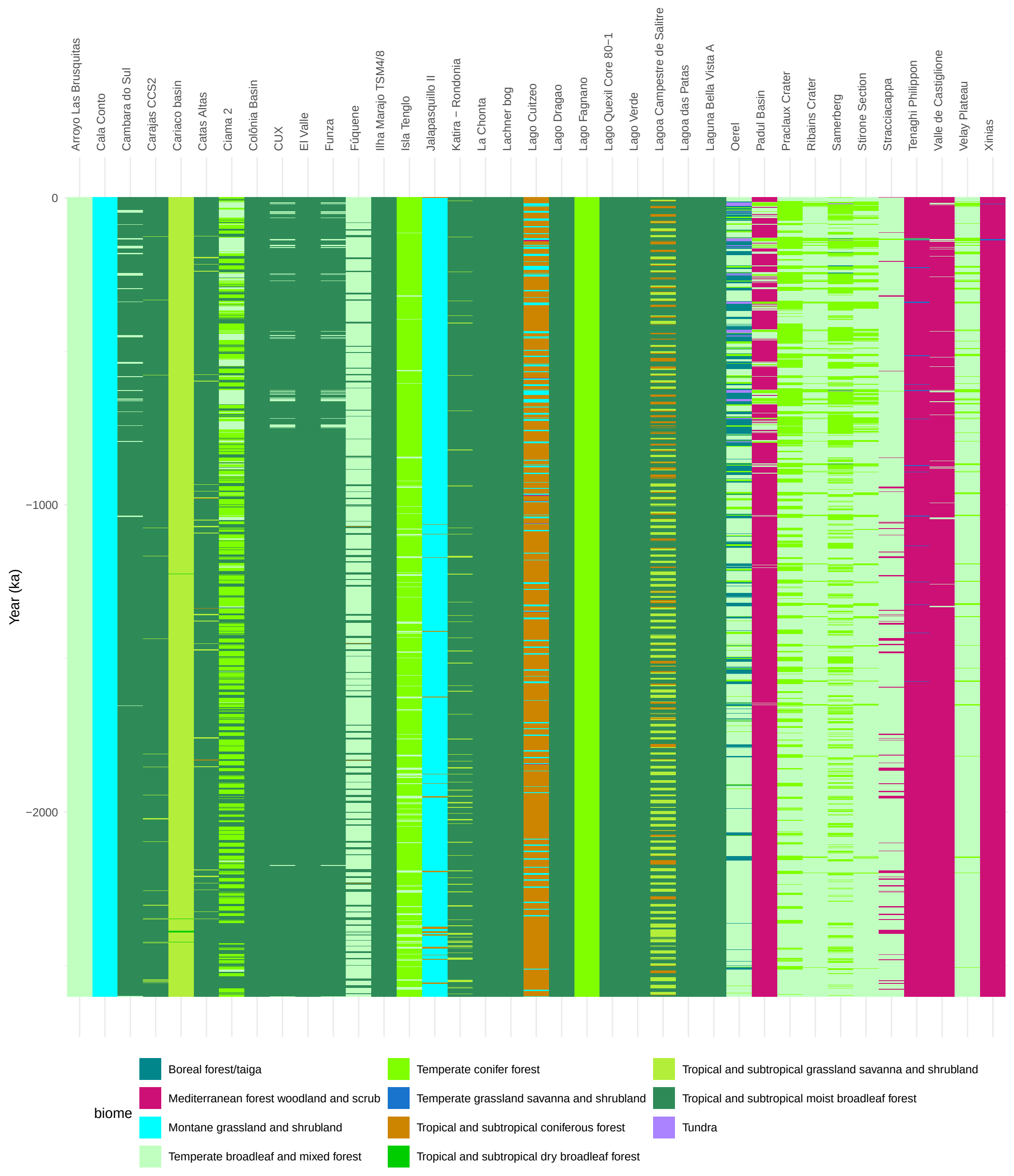

Figure S3: Biome types at drill core sites from Hooghiemstra et al. (2022), part 2.

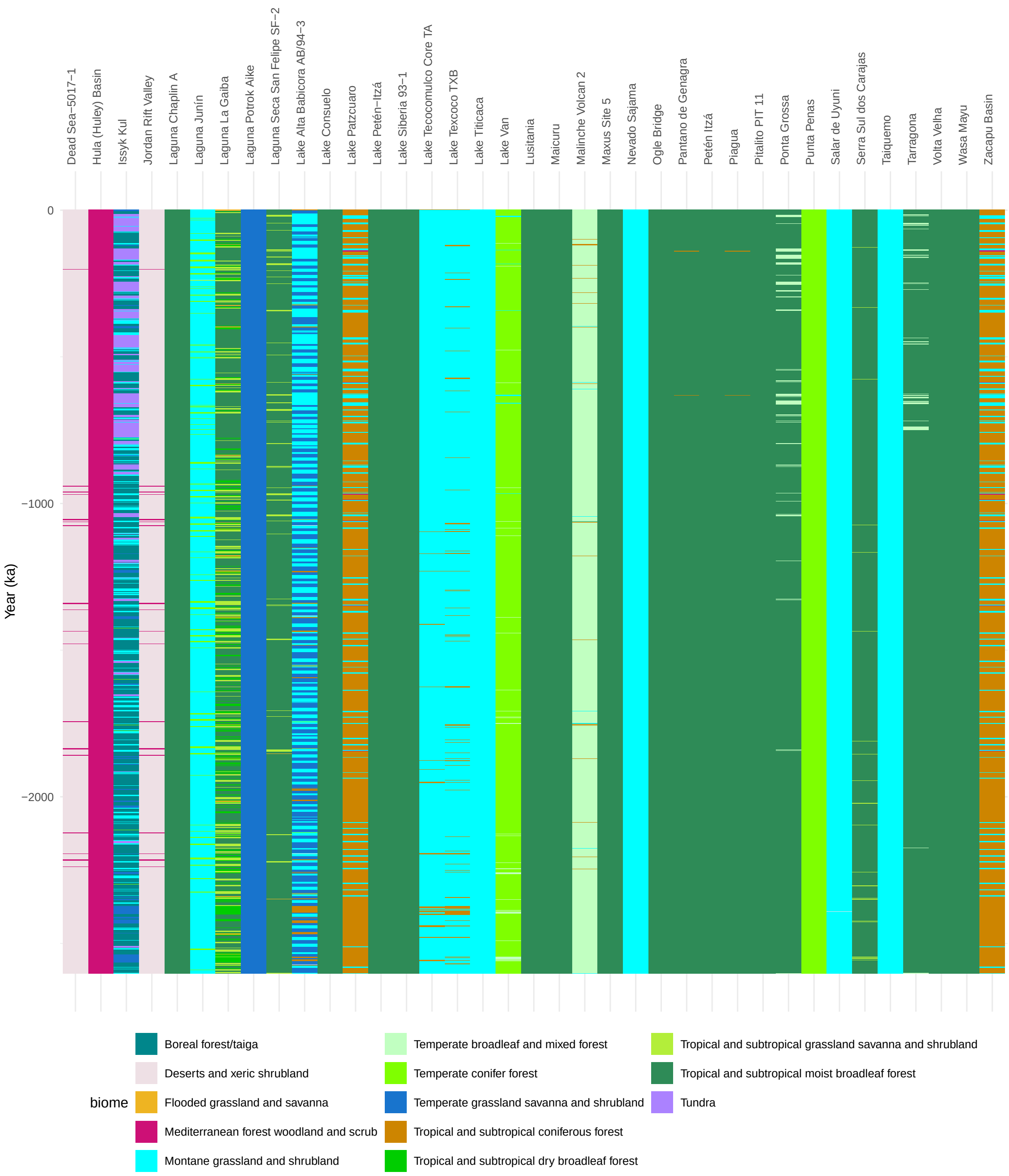

Figure S4: Biome types at drill core sites from Hooghiemstra et al. (2022), part 3.

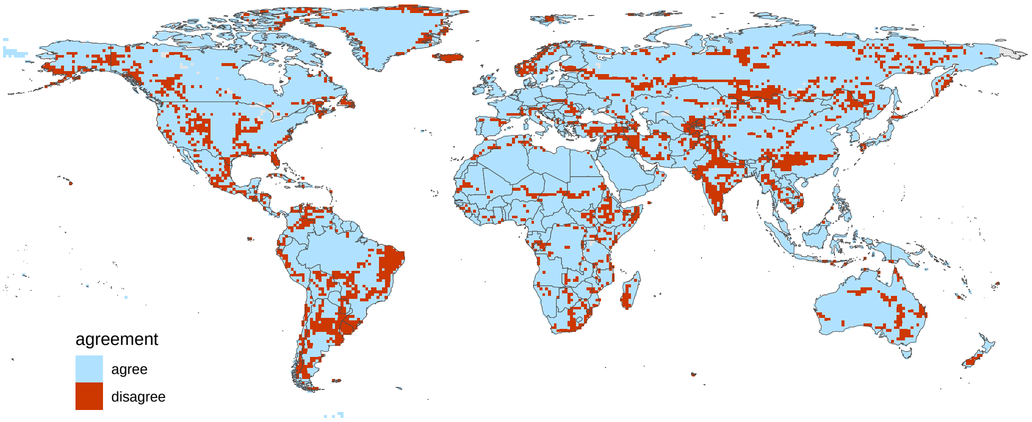

Figure S5: Agreement of observation-based and modeled biome distributions. For this map, observation-based and modeled biomes from Figs 1a, b were used.

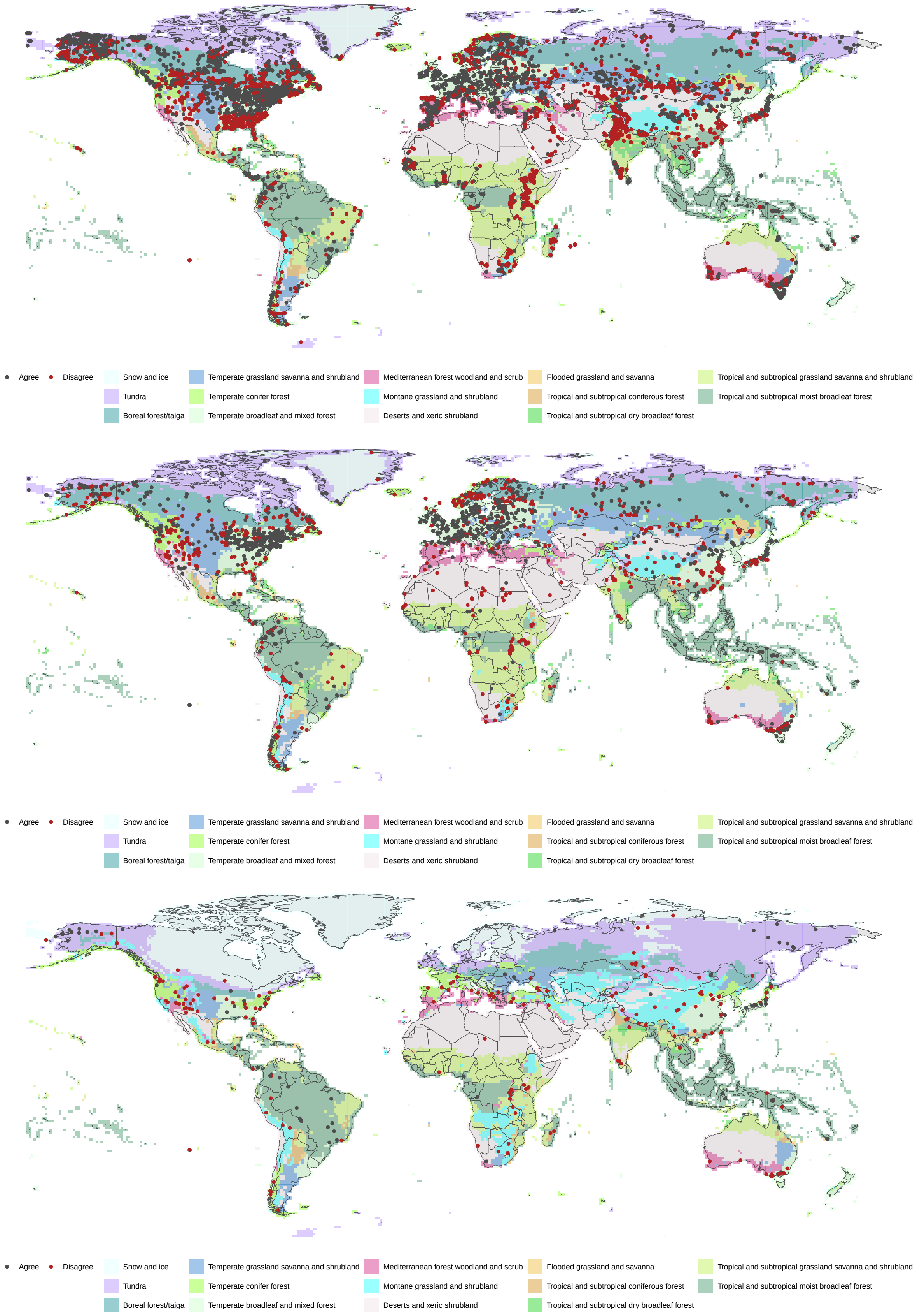

Figure S6: Comparison of modeled biomes and the BIOME6000 data. Figures show comparisons for 0 ka, 6 ka and 21 ka. For the three time periods, 57.5% of 9109 sites, 59.8% of 1815 sites and 36.4% of 283 sites of the modeled sites agree with reconstructed biome type at those sites.

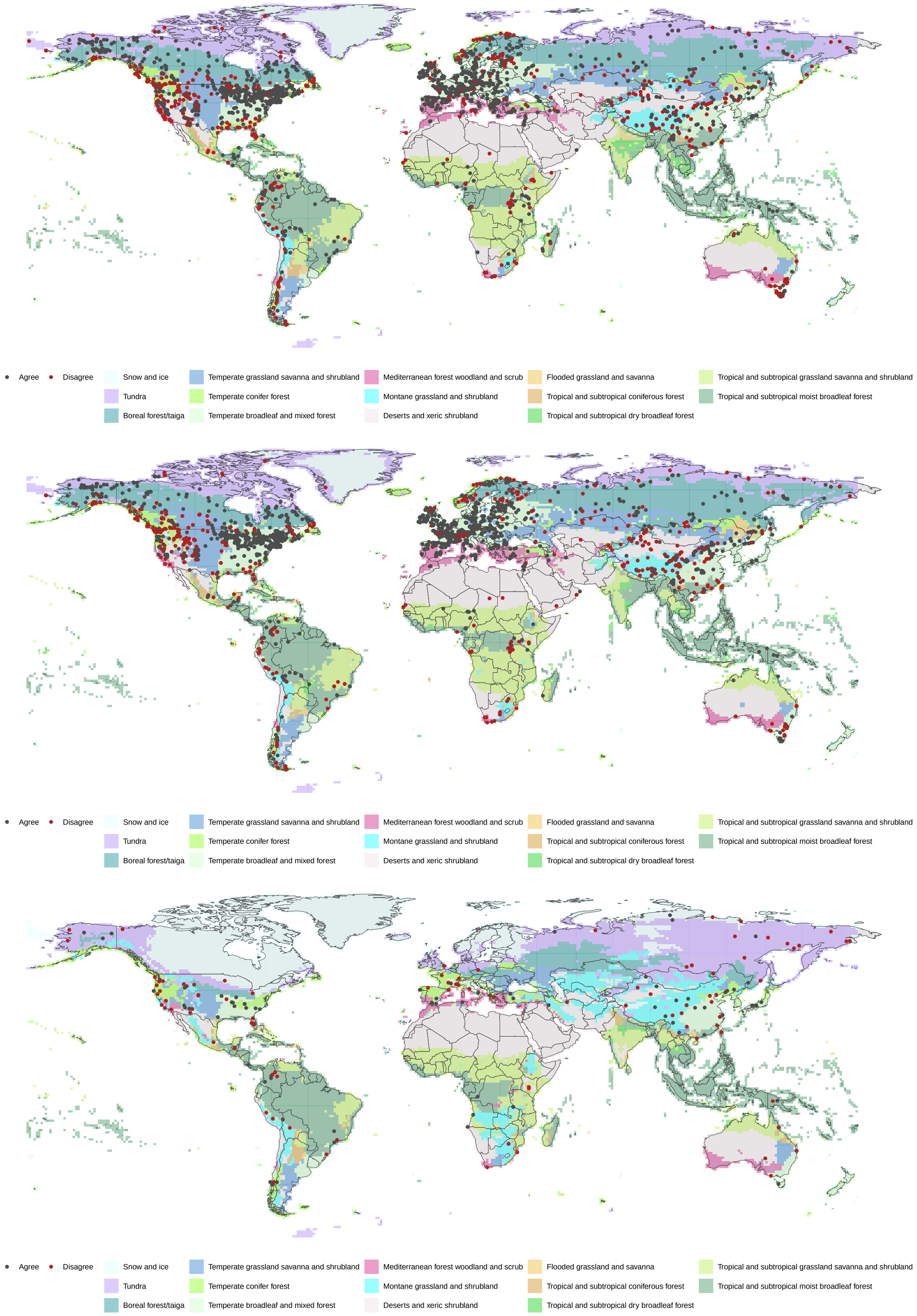

Figure S7: Comparison of modeled biomes and the LegacyPollen2.0 data. Figures show comparisons for 0 ka, 6 ka and 21 ka. For the three time periods, 71.8% of 2234 sites, 72.6% of 1997 sites and 43.6% of 188 sites of the modeled sites agree with reconstructed biome type at those sites.

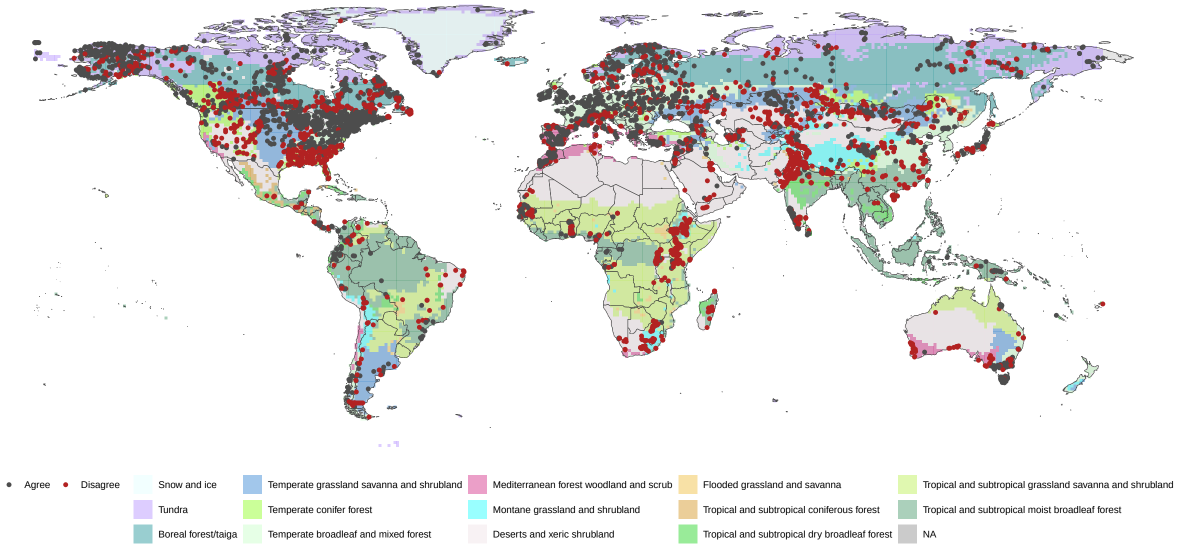

Figure S8: Comparison of the Olson et al. (2001) biomes and pollen-derived biomes for 0 ka. Biomes agreed for 70.6 of 2234 sites for LegacyPollen2.0 and for 57.1% of 9109 sites for BIOME6000.

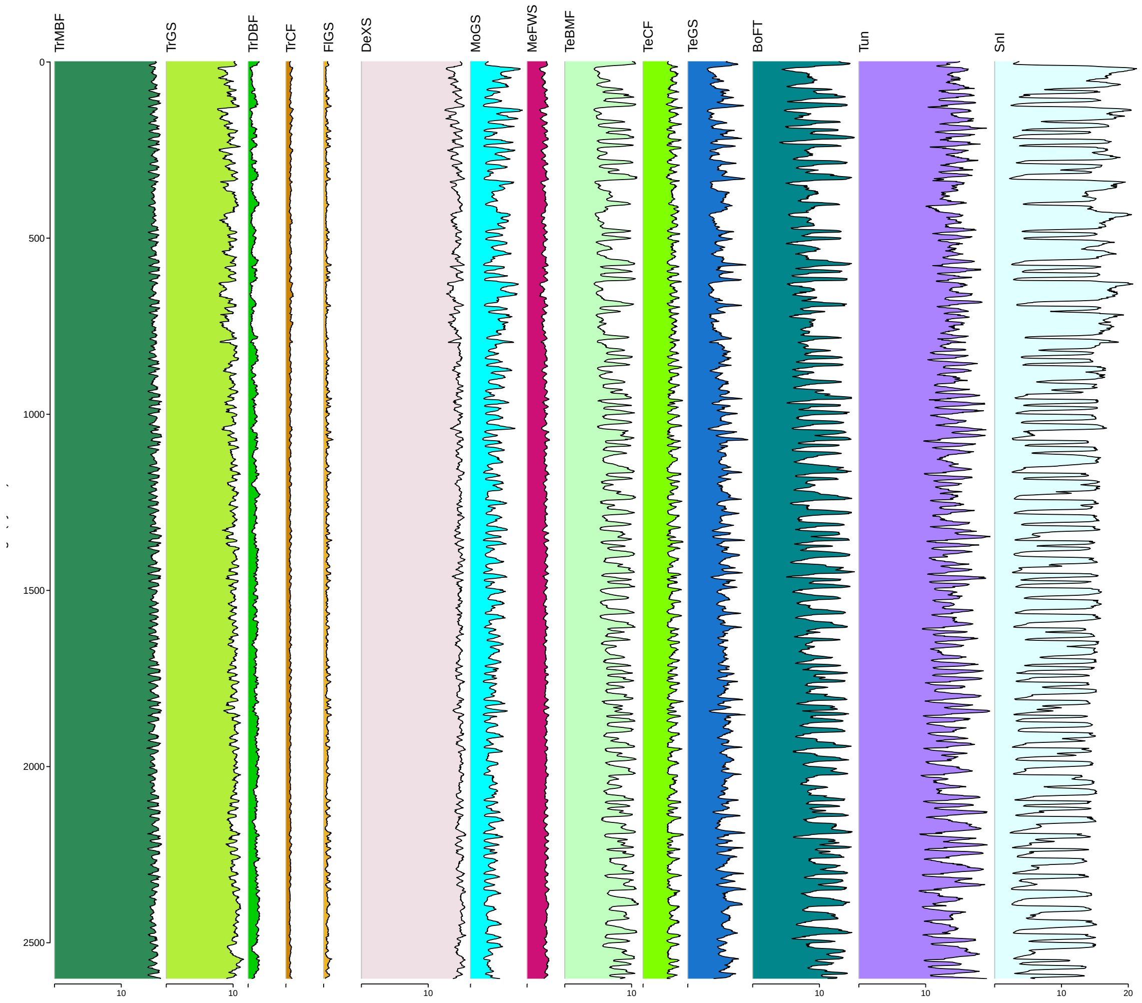

Figure S9: Time series of the fractional cover of different biome types. Abbreviations of biome types are defined in Table 1. In contrast to Fig. 2, x-axes are scaled between zero and the maximum cover fraction per biome.

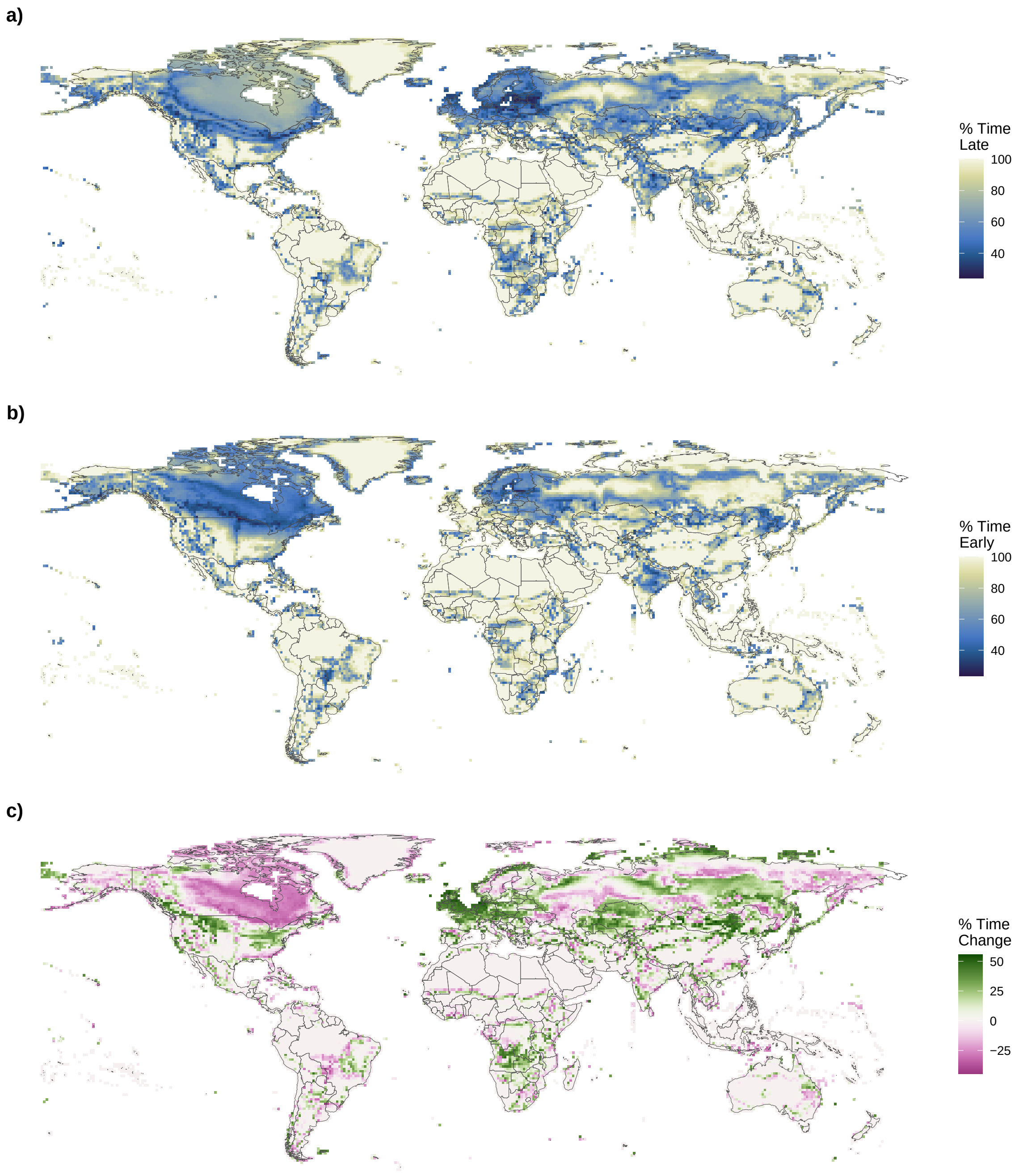

Figure S10: Hotspots of biome change during the last and first 150 ka of the study period. The maps show the percent of time periods grid cells were covered by the most frequent biome type in the (a) first and (b) last 150 ka of the study period. Here, 100 indicates that biomes were stable and covered by the same biome type during the entire study periods whereas lower values (blue) indicate hotspots of biome change. Panel (c) shows changes between late and early stability values, i.e., purple represents hotspots of biome change and less stability stability in the early Quaternary, green hotspots of biome change and low stability in the late Quaternary.

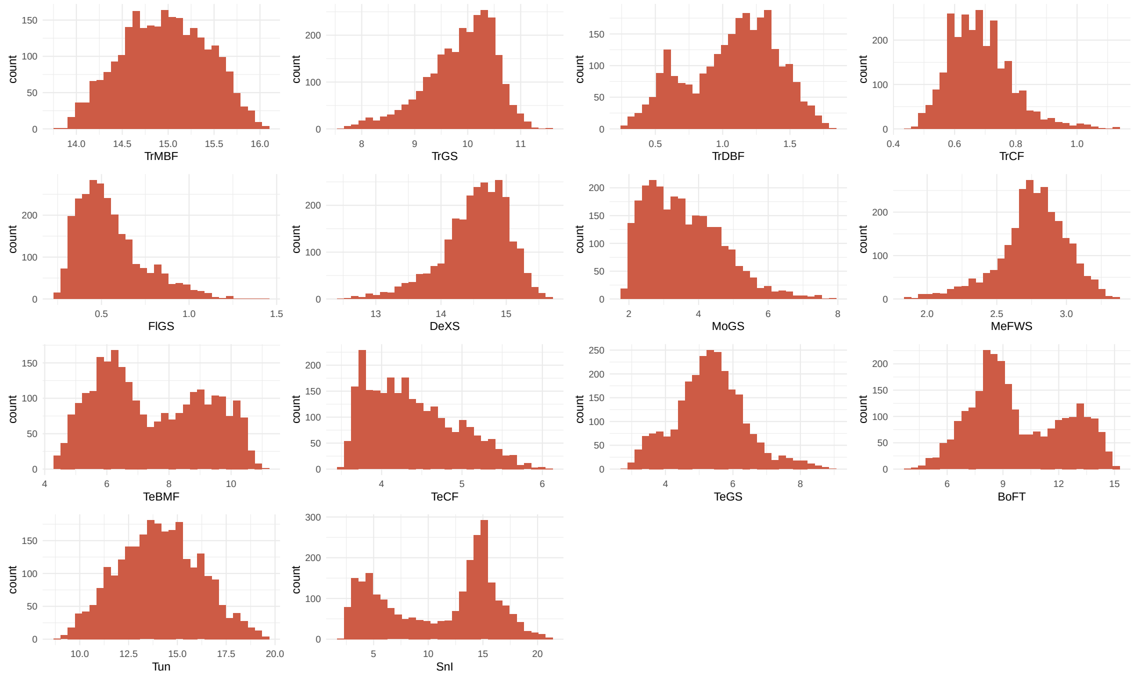

Figure S11: Histograms of biome cover for entire study period. Histograms were created by using the percent coverage of each biome in each time step of the modeled period. Note that ranges of both axes differ between panels due to substantial differences between coverage of the modeled biomes. Abbreviations of biome types are defined in Table 1. For each histogram, 30 equidistant bins ranging between minimum and maximum values of the respective biome type were used; accordingly the ranges of bins differs between biome.

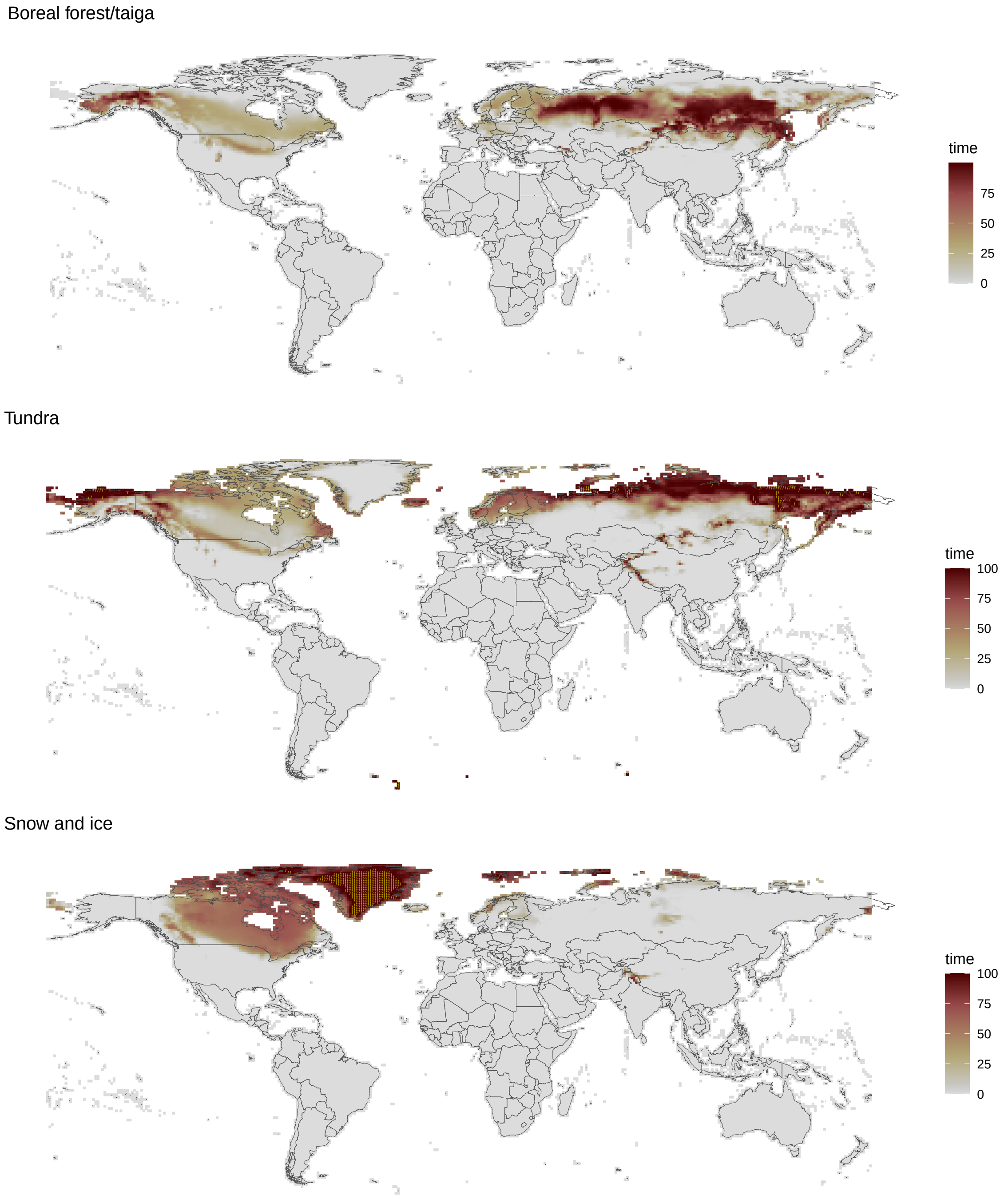

Figure S12: Percent time covered by biome during entire study period. Yellow hatching indicates areas where the biome was present during the entire study period.

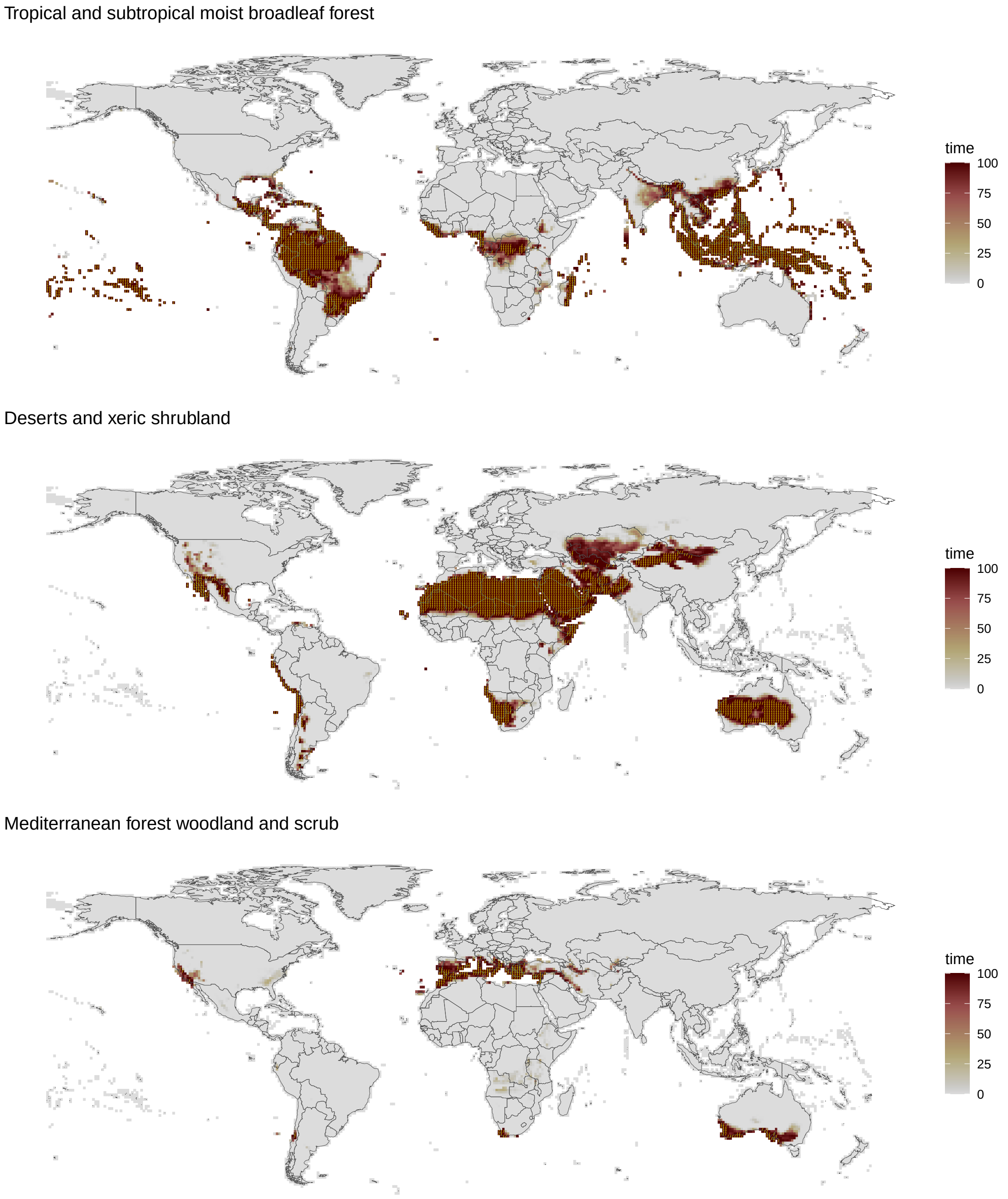

Figure S13: Percent time covered by biome during entire study period. Yellow hatching indicates areas where the biome was present during the entire study period.

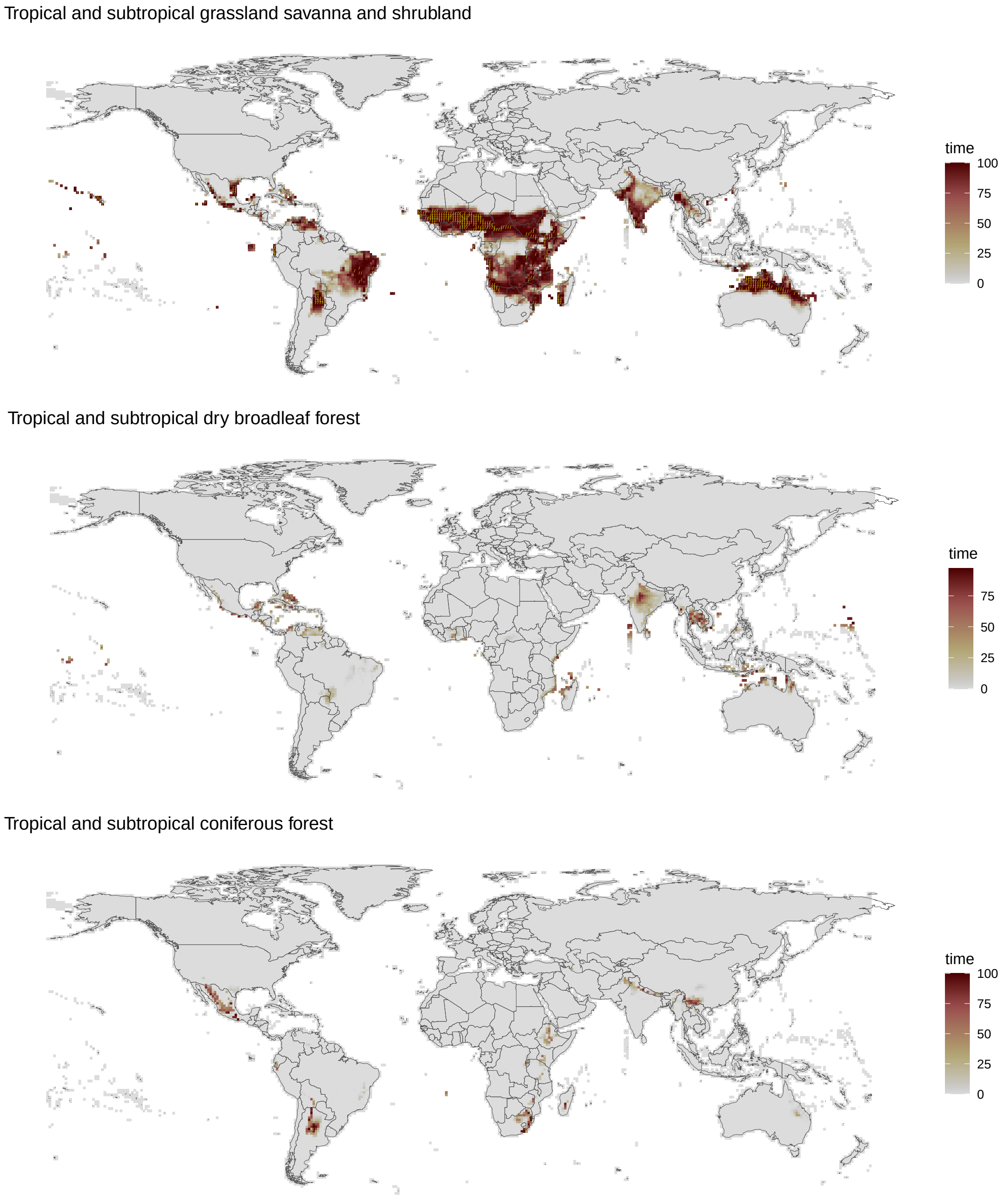

Figure S14: Percent time covered by biome during entire study period. Yellow hatching indicates areas where the biome was present during the entire study period.

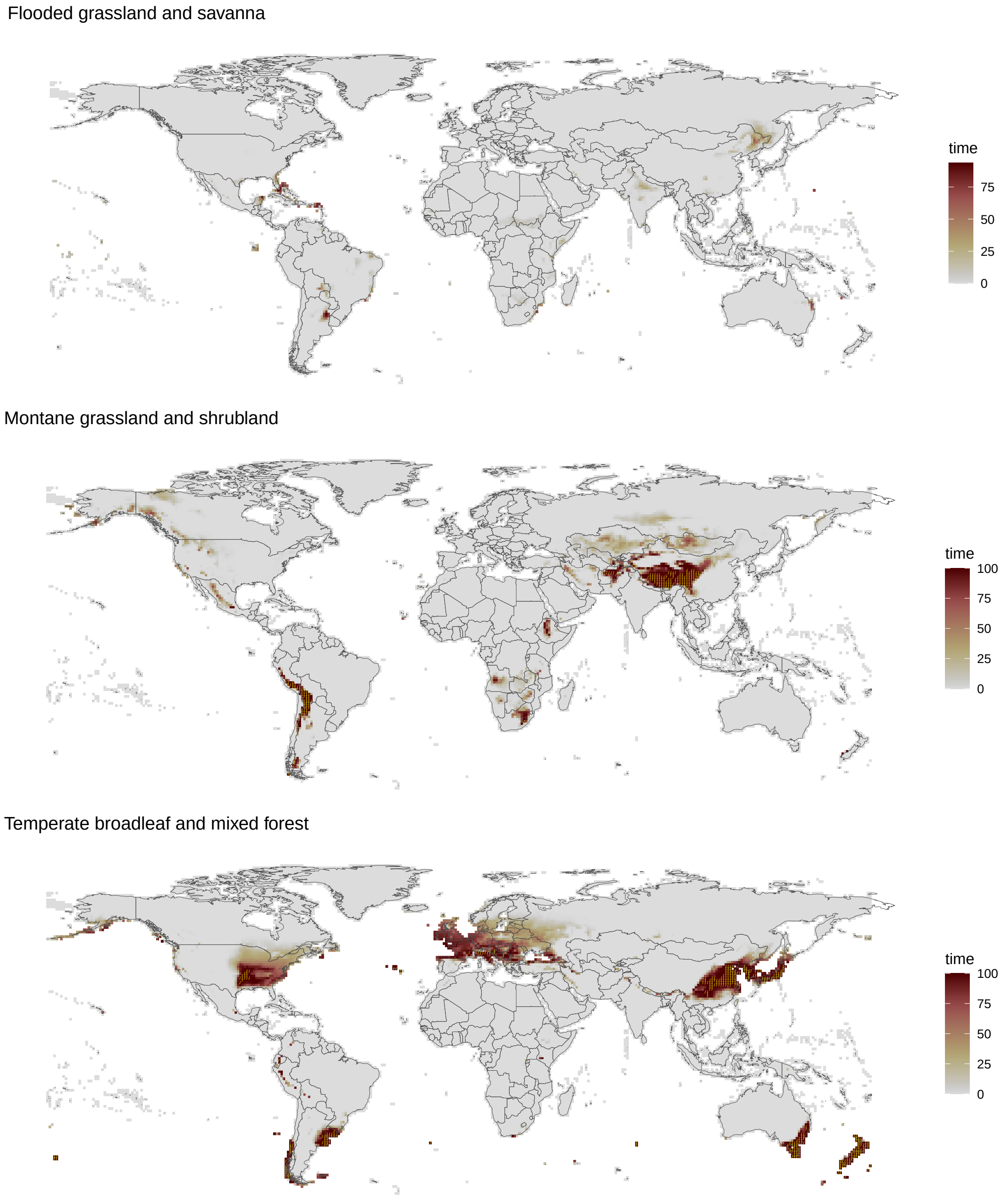

Figure S15: Percent time covered by biome during entire study period. Yellow hatching indicates areas where the biome was present during the entire study period.

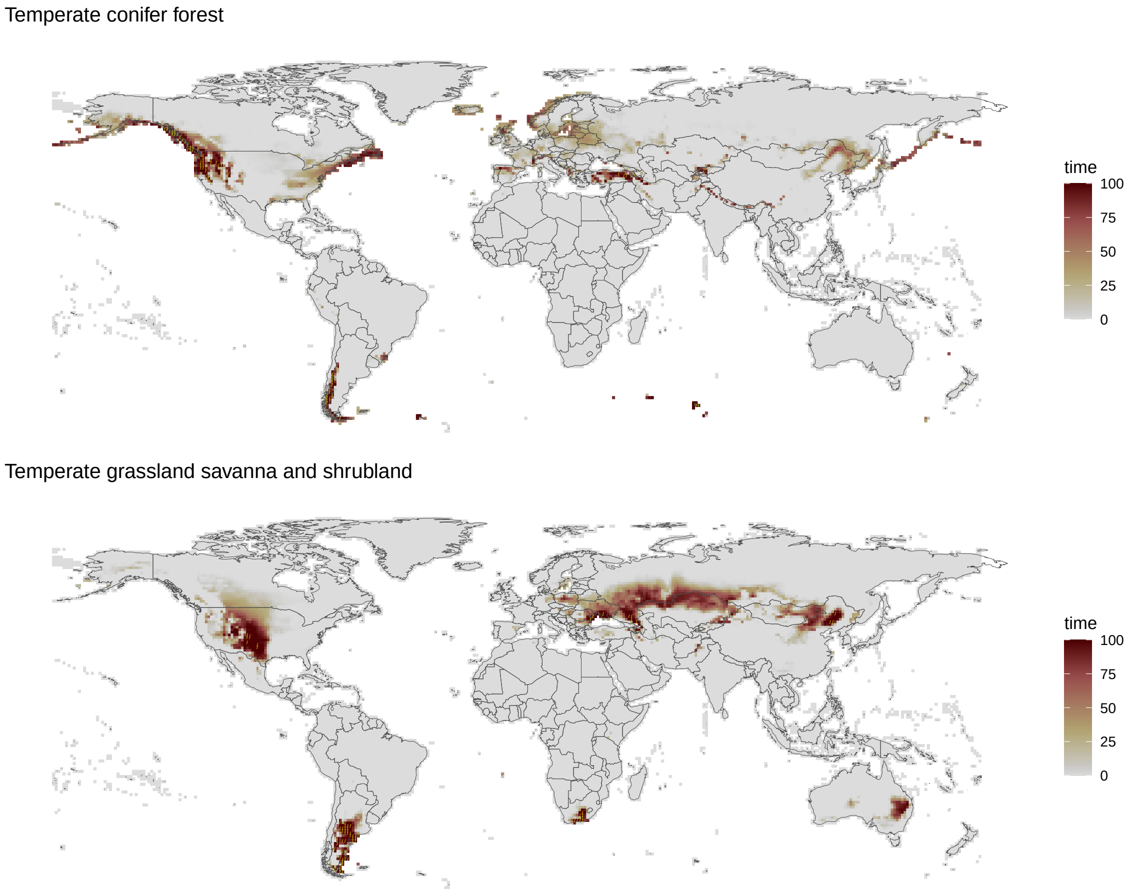

Figure S16: Percent time covered by biome during entire study period. Yellow hatching indicates areas where the biome was present during the entire study period.

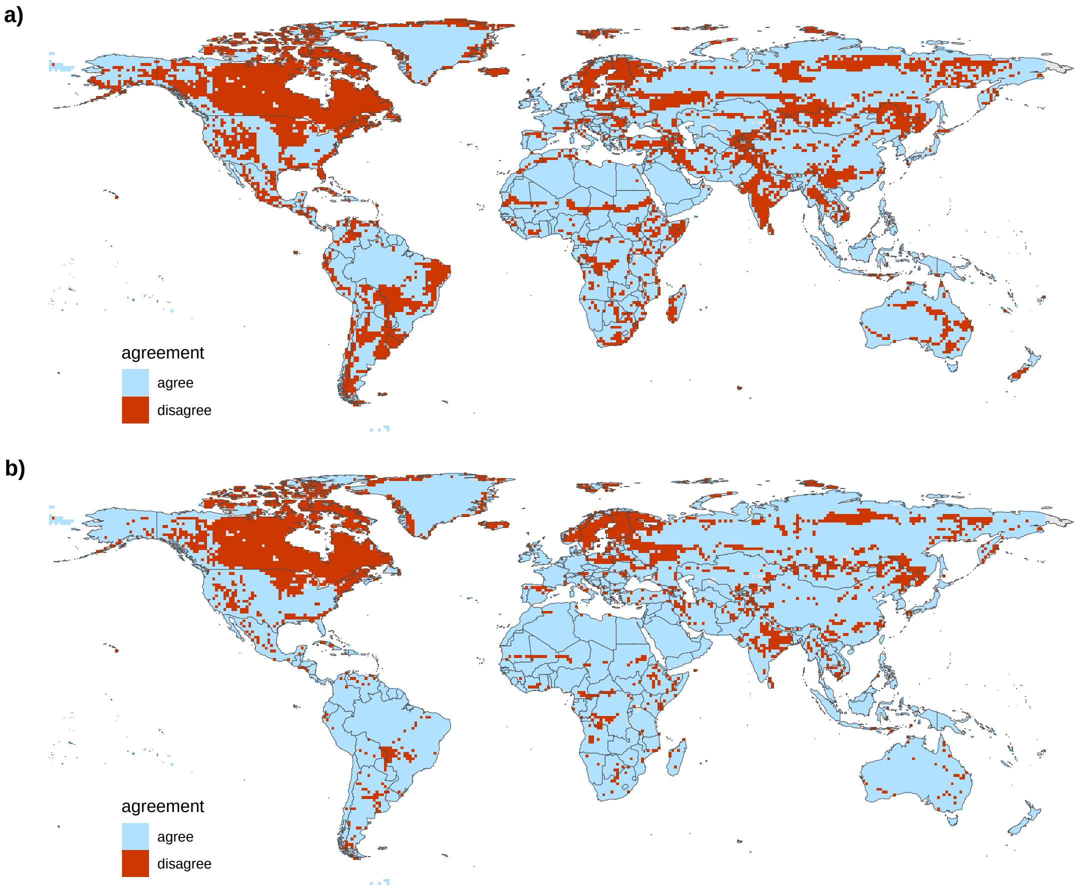

Figure S17: Agreement between the current biome distribution and the most frequent biome distribution for the entire study period. For the comparison, the observation-based biome map (panel a, Fig. 1a) and the modeled biome map (panel b, Fig. 1b) were used. The most frequent biome distribution is provided in Fig. 3a.

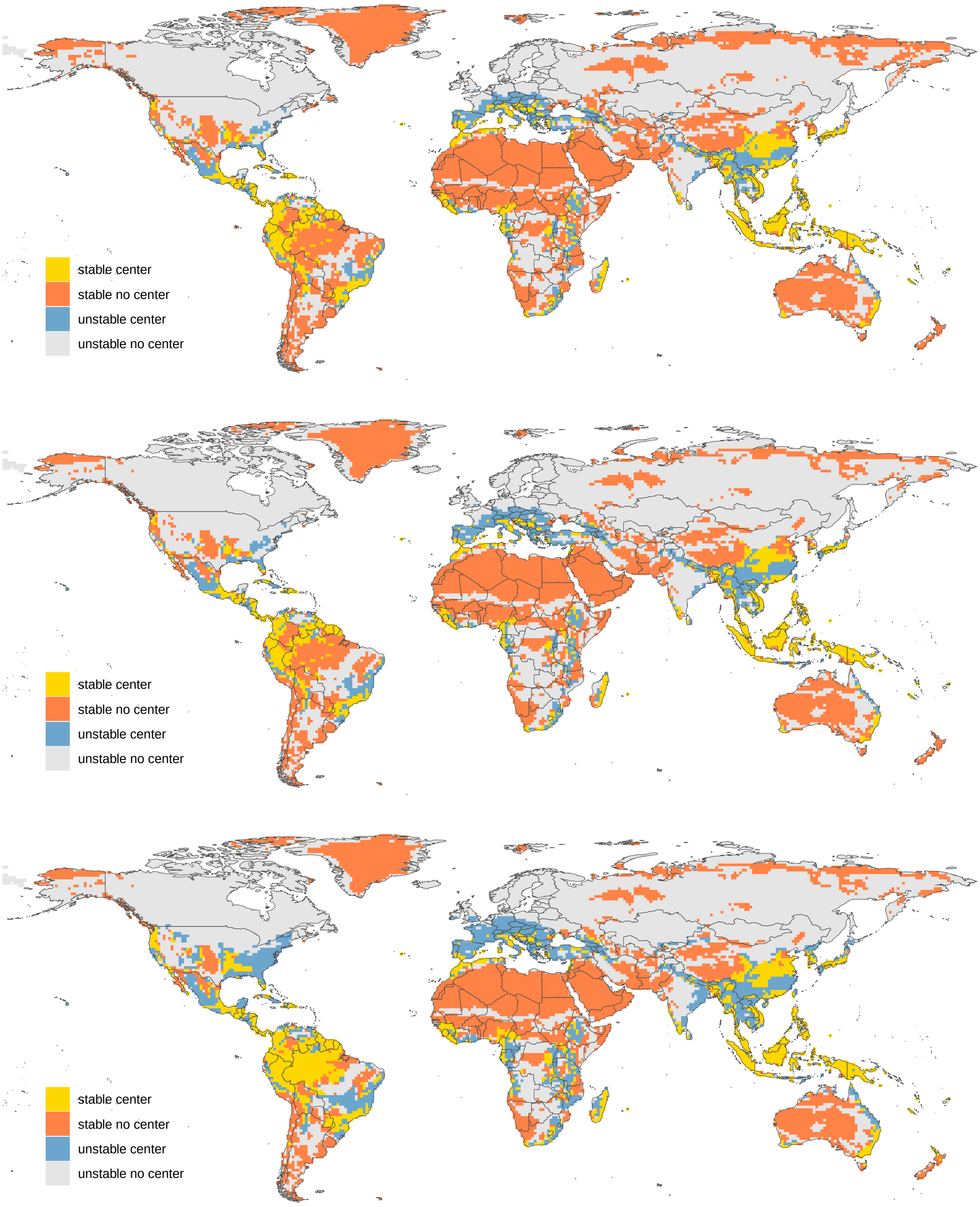

Figure S18: Overlap between areas with biome stability. In the main text, a grid cell was classified as stable when it was covered by the same biome type for 100% of the time period, and as center of species richness when more than 1765 species were present (following Cai et al. 2023). Here, a grid cell was considered stable if the same biome type occurred during 90% (top) and 95% (middle, bottom) of the study period and as centers of species richness when more than 1765 (top, middle) or 1500 species (bottom occurred. In the legend, 'stable' and 'unstable' refer to biome stability, 'center' and 'no center' refer to centers of species richness.

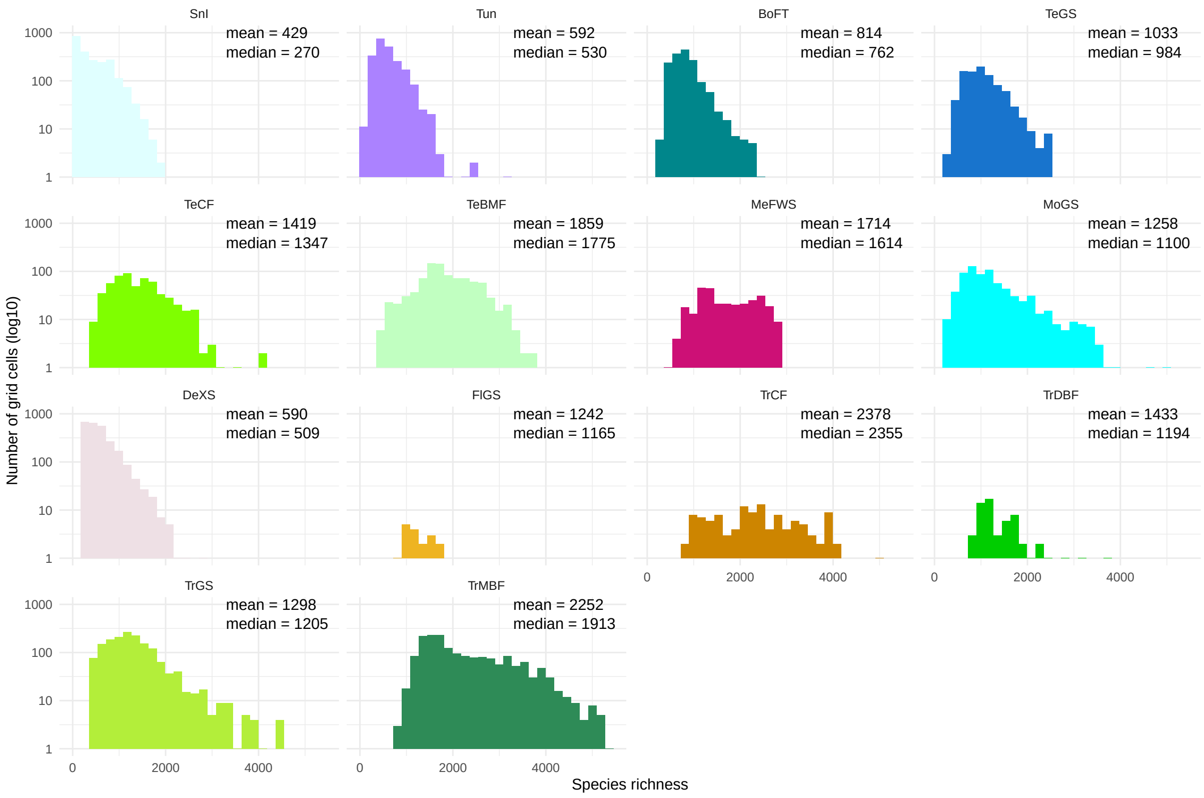

Figure S19: Biome-specific species richness. Histograms represent the number of grid cells with species richness in different bins. Here, 30 bins were used. The number of grid cells is on a log scale. ‘mean’ and ‘median’ are the mean and median values of species richness per biome.

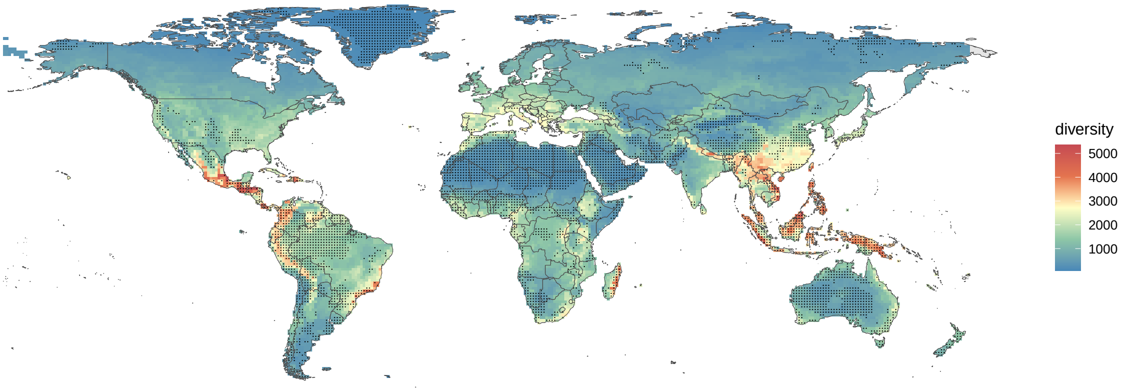

Figure S20: Species richness and biome stability. Colors represent species richness in the Cai et al. (2023) data, black points indicate biome stability according to our analysis.

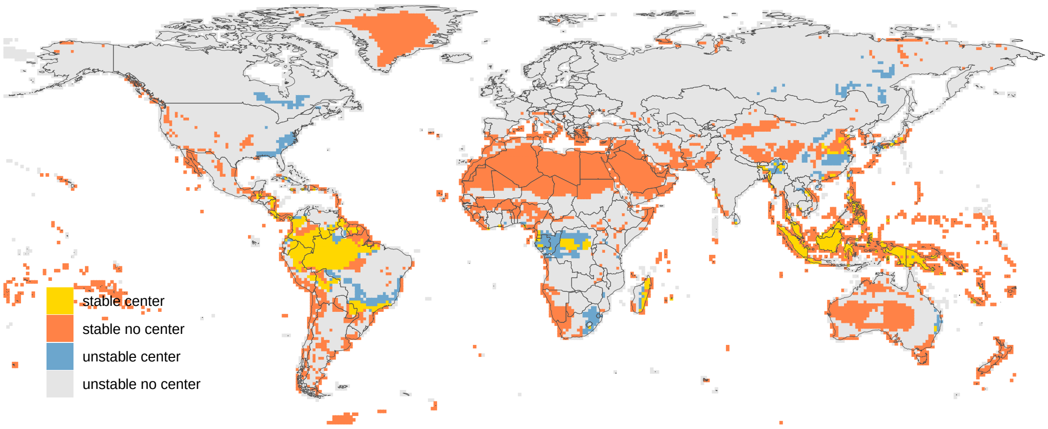

Figure S21: Overlap between areas with biome stability during the entire study period and centers of plant species richness according to Sabatini et al. (2021). In the legend, 'stable' and 'unstable' refer to biome stability (areas with value =100 in Fig. **??** are stable, areas with value <100 are unstable), 'center' and 'no center' refer to centers of species richness.

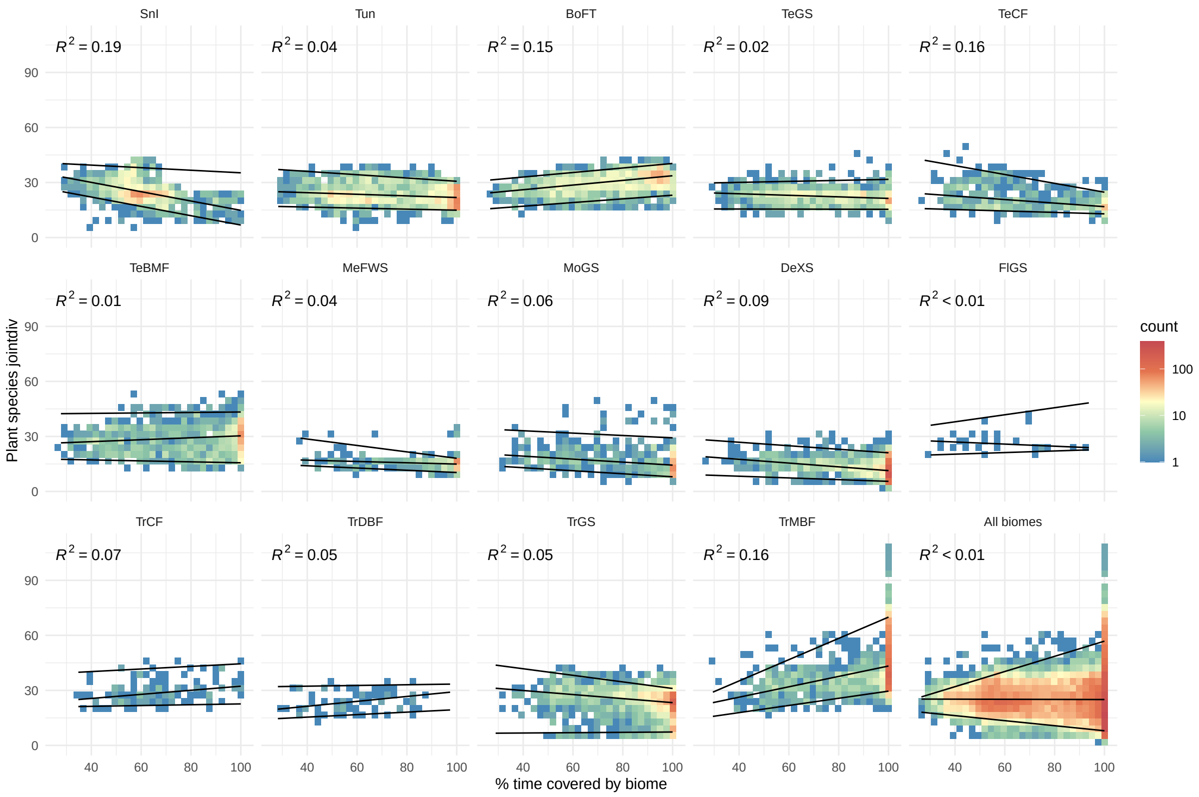

Figure S22: Relationship between diversity and stability per biome. Plant species richness was taken from Sabatini et al. (2021), % time covered by biome represents biome stability. Colors represent the number of grid cell in each bin defined by stability and species richness. Count values are on a log scale. Lines represent quantile regressions for the 5, 50 and 95% percentiles. Pseudo R^2 are provided for the 50% percentile regression models.
