# Supplementary material for: The Effects of Biome Stability During the Quaternary on Plant Diversity
Source: Ecol Evol. 2026 Jun 30;16(7):e73884. doi: 10.1002/ece3.73884 (PMC13316459; doi:10.1002/ece3.73884)
Supplement: Supplementary file 2 — Data S1: Biome types for drill core sites, sites taken from Hooghiemstra et al. (2022) Scientific drilling, Table S1. [file ECE3-16-e73884-s003.pdf]

Supplement S2 - Biome types for drill core sites, sites taken from Hooghiemstra et al. (2022) Scientific drilling, Supplementary Table S1

Biome types were extracted for the 1° grid cell from the model results that contains the respective site. Duplicates, i.e., if several sites are within one 1° grid cell, were removed.

For each site, the number of different biome types at the site during the Quaternary, the biome type modeled under current conditions, and the most frequent biome types (up to 3) are provided.

Time series are provided in supplementary figures.

```
----------- Arroyo Las Brusquitas
Number of biomes:           1
Current biome in model:     Temperate broadleaf and mixed forest
Most common biome type:     Temperate broadleaf and mixed forest, 2605 occurrences

----------- Cala Conto
Number of biomes:           1
Current biome in model:     Montane grassland and shrubland
Most common biome type:     Montane grassland and shrubland, 2605 occurrences

----------- Cambara do Sul
Number of biomes:           2
Current biome in model:     Tropical and subtropical moist broadleaf forest
1st most common biome type: Tropical and subtropical moist broadleaf forest, 2535 occurrences
2nd most common biome type: Temperate broadleaf and mixed forest, 70 occurrences

----------- Carajas CCS2
Number of biomes:           2
Current biome in model:     Tropical and subtropical moist broadleaf forest
1st most common biome type: Tropical and subtropical moist broadleaf forest, 2576 occurrences
2nd most common biome type: Tropical and subtropical grassland savanna and shrubland, 29 occurrences

----------- Cariaco basin
Number of biomes:           2
Current biome in model:     Tropical and subtropical grassland savanna and shrubland
1st most common biome type: Tropical and subtropical grassland savanna and shrubland, 2597 occurrences
2nd most common biome type: Tropical and subtropical dry broadleaf forest, 8 occurrences

----------- Catas Altas
Number of biomes:           3
Current biome in model:     Tropical and subtropical moist broadleaf forest
1st most common biome type: Tropical and subtropical moist broadleaf forest, 2550 occurrences
2nd most common biome type: Tropical and subtropical grassland savanna and shrubland, 50 occurrences
3nd most common biome type: Tropical and subtropical coniferous forest, 5 occurrences

----------- Ciama 2
Number of biomes:           3
Current biome in model:     Temperate conifer forest
1st most common biome type: Temperate conifer forest, 1327 occurrences
2nd most common biome type: Tropical and subtropical moist broadleaf forest, 940 occurrences
3nd most common biome type: Temperate broadleaf and mixed forest, 338 occurrences

----------- Colônia Basin
Number of biomes:           1
Current biome in model:     Tropical and subtropical moist broadleaf forest
Most common biome type:     Tropical and subtropical moist broadleaf forest, 2605 occurrences

----------- CUX
Number of biomes:           2
Current biome in model:     Tropical and subtropical moist broadleaf forest
1st most common biome type: Tropical and subtropical moist broadleaf forest, 2546 occurrences
2nd most common biome type: Temperate broadleaf and mixed forest, 59 occurrences

----------- Dead Sea-5017-1
Number of biomes:           2
Current biome in model:     Deserts and xeric shrubland
1st most common biome type: Deserts and xeric shrubland, 2567 occurrences
2nd most common biome type: Mediterranean forest woodland and scrub, 38 occurrences

----------- El Valle
Number of biomes:           1
Current biome in model:     Tropical and subtropical moist broadleaf forest
Most common biome type:     Tropical and subtropical moist broadleaf forest, 2605 occurrences

----------- Funza
Number of biomes:           2
Current biome in model:     Tropical and subtropical moist broadleaf forest
1st most common biome type: Tropical and subtropical moist broadleaf forest, 2546 occurrences
2nd most common biome type: Temperate broadleaf and mixed forest, 59 occurrences
```

```
----------- Fúquene
Number of biomes:           3
Current biome in model:     Temperate broadleaf and mixed forest
1st most common biome type: Temperate broadleaf and mixed forest, 2202 occurrences
2nd most common biome type: Tropical and subtropical moist broadleaf forest, 398 occurrences
3nd most common biome type: Tropical and subtropical coniferous forest, 5 occurrences

----------- Füramoos
Number of biomes:           3
Current biome in model:     Temperate broadleaf and mixed forest
1st most common biome type: Temperate broadleaf and mixed forest, 2395 occurrences
2nd most common biome type: Temperate conifer forest, 197 occurrences
3nd most common biome type: Boreal forest/taiga, 13 occurrences

----------- Grande Pile
Number of biomes:           2
Current biome in model:     Temperate broadleaf and mixed forest
1st most common biome type: Temperate broadleaf and mixed forest, 1543 occurrences
2nd most common biome type: Temperate conifer forest, 1062 occurrences

----------- Horoszki Duze
Number of biomes:           7
Current biome in model:     Temperate broadleaf and mixed forest
1st most common biome type: Temperate grassland savanna and shrubland, 1447 occurrences
2nd most common biome type: Temperate broadleaf and mixed forest, 588 occurrences
3nd most common biome type: Temperate conifer forest, 516 occurrences

----------- Hula (Huley) Basin
Number of biomes:           1
Current biome in model:     Mediterranean forest woodland and scrub
Most common biome type:     Mediterranean forest woodland and scrub, 2605 occurrences

----------- Ilha Marajo TSM4/8
Number of biomes:           1
Current biome in model:     Tropical and subtropical moist broadleaf forest
Most common biome type:     Tropical and subtropical moist broadleaf forest, 2605 occurrences

----------- Ioannina
Number of biomes:           3
Current biome in model:     Mediterranean forest woodland and scrub
1st most common biome type: Mediterranean forest woodland and scrub, 2177 occurrences
2nd most common biome type: Temperate broadleaf and mixed forest, 337 occurrences
3nd most common biome type: Temperate conifer forest, 91 occurrences

----------- Isla Tenglo
Number of biomes:           2
Current biome in model:     Temperate conifer forest
1st most common biome type: Temperate conifer forest, 2319 occurrences
2nd most common biome type: Temperate broadleaf and mixed forest, 286 occurrences

----------- Issyk Kul
Number of biomes:           5
Current biome in model:     Temperate grassland savanna and shrubland
1st most common biome type: Boreal forest/taiga, 1428 occurrences
2nd most common biome type: Montane grassland and shrubland, 524 occurrences
3nd most common biome type: Tundra, 464 occurrences

----------- Jalapasquillo II
Number of biomes:           2
Current biome in model:     Tropical and subtropical coniferous forest
1st most common biome type: Montane grassland and shrubland, 2539 occurrences
2nd most common biome type: Tropical and subtropical coniferous forest, 66 occurrences

----------- Jordan Rift Valley
Number of biomes:           2
Current biome in model:     Deserts and xeric shrubland
1st most common biome type: Deserts and xeric shrubland, 2567 occurrences
2nd most common biome type: Mediterranean forest woodland and scrub, 38 occurrences

----------- Katira - Rondonia
Number of biomes:           2
Current biome in model:     Tropical and subtropical moist broadleaf forest
1st most common biome type: Tropical and subtropical moist broadleaf forest, 2489 occurrences
2nd most common biome type: Tropical and subtropical grassland savanna and shrubland, 116 occurrences

----------- La Chonta
Number of biomes:           1
Current biome in model:     Tropical and subtropical moist broadleaf forest
Most common biome type:     Tropical and subtropical moist broadleaf forest, 2605 occurrences

----------- Lac du Bouchet
Number of biomes:           2
Current biome in model:     Temperate broadleaf and mixed forest
1st most common biome type: Temperate broadleaf and mixed forest, 1635 occurrences
2nd most common biome type: Temperate conifer forest, 970 occurrences
```

```
----------- Lachner bog
Number of biomes:            1
Current biome in model:      Tropical and subtropical moist broadleaf forest
Most common biome type:      Tropical and subtropical moist broadleaf forest, 2605 occurrences

----------- Lago Cuitzeo
Number of biomes:            3
Current biome in model:      Tropical and subtropical coniferous forest
1st most common biome type: Tropical and subtropical coniferous forest, 2273 occurrences
2nd most common biome type: Montane grassland and shrubland, 322 occurrences
3nd most common biome type: Mediterranean forest woodland and scrub, 10 occurrences

----------- Lago di Vico
Number of biomes:            3
Current biome in model:      Mediterranean forest woodland and scrub
1st most common biome type: Temperate broadleaf and mixed forest, 2385 occurrences
2nd most common biome type: Mediterranean forest woodland and scrub, 216 occurrences
3nd most common biome type: Temperate conifer forest, 4 occurrences

----------- Lago Dragao
Number of biomes:            1
Current biome in model:      Tropical and subtropical moist broadleaf forest
Most common biome type:      Tropical and subtropical moist broadleaf forest, 2605 occurrences

----------- Lago Fagnano
Number of biomes:            1
Current biome in model:      Temperate conifer forest
Most common biome type:      Temperate conifer forest, 2605 occurrences

----------- Lago Grande di Monticchio
Number of biomes:            3
Current biome in model:      Mediterranean forest woodland and scrub
1st most common biome type: Mediterranean forest woodland and scrub, 1271 occurrences
2nd most common biome type: Temperate broadleaf and mixed forest, 1191 occurrences
3nd most common biome type: Temperate conifer forest, 143 occurrences

----------- Lago Quexil Core 80-1
Number of biomes:            1
Current biome in model:      Tropical and subtropical moist broadleaf forest
Most common biome type:      Tropical and subtropical moist broadleaf forest, 2605 occurrences

----------- Lago Verde
Number of biomes:            1
Current biome in model:      Tropical and subtropical moist broadleaf forest
Most common biome type:      Tropical and subtropical moist broadleaf forest, 2605 occurrences

----------- Lagoa Campestre de Salitre
Number of biomes:            3
Current biome in model:      Tropical and subtropical moist broadleaf forest
1st most common biome type: Tropical and subtropical moist broadleaf forest, 1657 occurrences
2nd most common biome type: Tropical and subtropical grassland savanna and shrubland, 668 occurrences
3nd most common biome type: Tropical and subtropical coniferous forest, 280 occurrences

----------- Lagoa das Patas
Number of biomes:            1
Current biome in model:      Tropical and subtropical moist broadleaf forest
Most common biome type:      Tropical and subtropical moist broadleaf forest, 2605 occurrences

----------- Laguna Bella Vista A
Number of biomes:            1
Current biome in model:      Tropical and subtropical moist broadleaf forest
Most common biome type:      Tropical and subtropical moist broadleaf forest, 2605 occurrences

----------- Laguna Chaplin A
Number of biomes:            1
Current biome in model:      Tropical and subtropical moist broadleaf forest
Most common biome type:      Tropical and subtropical moist broadleaf forest, 2605 occurrences

----------- Laguna Junín
Number of biomes:            3
Current biome in model:      Montane grassland and shrubland
1st most common biome type: Montane grassland and shrubland, 2435 occurrences
2nd most common biome type: Temperate conifer forest, 169 occurrences
3nd most common biome type: Temperate broadleaf and mixed forest, 1 occurrences

----------- Laguna La Gaiba
Number of biomes:            4
Current biome in model:      Flooded grassland and savanna
1st most common biome type: Tropical and subtropical moist broadleaf forest, 1768 occurrences
2nd most common biome type: Tropical and subtropical grassland savanna and shrubland, 443 occurrences
3nd most common biome type: Tropical and subtropical dry broadleaf forest, 357 occurrences

----------- Laguna Potrok Aike
Number of biomes:            1
Current biome in model:      Temperate grassland savanna and shrubland
Most common biome type:      Temperate grassland savanna and shrubland, 2605 occurrences
```

```
----------- Laguna Seca San Felipe SF-2
Number of biomes:            3
Current biome in model:      Tropical and subtropical moist broadleaf forest
1st most common biome type: Tropical and subtropical moist broadleaf forest, 2515 occurrences
2nd most common biome type: Tropical and subtropical grassland savanna and shrubland, 88 occurrences
3nd most common biome type: Flooded grassland and savanna, 2 occurrences

----------- Lake Albert
Number of biomes:            4
Current biome in model:      Tropical and subtropical grassland savanna and shrubland
1st most common biome type: Tropical and subtropical grassland savanna and shrubland, 1719 occurrences
2nd most common biome type: Tropical and subtropical moist broadleaf forest, 866 occurrences
3nd most common biome type: Flooded grassland and savanna, 19 occurrences

----------- Lake Alta Babicora AB/94-3
Number of biomes:            5
Current biome in model:      Tropical and subtropical coniferous forest
1st most common biome type: Montane grassland and shrubland, 1234 occurrences
2nd most common biome type: Temperate grassland savanna and shrubland, 1211 occurrences
3nd most common biome type: Tropical and subtropical coniferous forest, 143 occurrences

----------- Lake Baikal
Number of biomes:            4
Current biome in model:      Boreal forest/taiga
1st most common biome type: Boreal forest/taiga, 1708 occurrences
2nd most common biome type: Montane grassland and shrubland, 647 occurrences
3nd most common biome type: Tundra, 211 occurrences

----------- Lake Bambili
Number of biomes:            1
Current biome in model:      Tropical and subtropical moist broadleaf forest
Most common biome type:      Tropical and subtropical moist broadleaf forest, 2605 occurrences

----------- Lake Banyoles
Number of biomes:            3
Current biome in model:      Temperate broadleaf and mixed forest
1st most common biome type: Temperate broadleaf and mixed forest, 2103 occurrences
2nd most common biome type: Temperate conifer forest, 483 occurrences
3nd most common biome type: Mediterranean forest woodland and scrub, 19 occurrences

----------- Lake Barombi-Mbo Crater
Number of biomes:            1
Current biome in model:      Tropical and subtropical moist broadleaf forest
Most common biome type:      Tropical and subtropical moist broadleaf forest, 2605 occurrences

----------- Lake Biwa
Number of biomes:            2
Current biome in model:      Temperate broadleaf and mixed forest
1st most common biome type: Temperate broadleaf and mixed forest, 2557 occurrences
2nd most common biome type: Tropical and subtropical moist broadleaf forest, 48 occurrences

----------- Lake Bosumtwi
Number of biomes:            3
Current biome in model:      Tropical and subtropical moist broadleaf forest
1st most common biome type: Tropical and subtropical moist broadleaf forest, 1587 occurrences
2nd most common biome type: Tropical and subtropical grassland savanna and shrubland, 1007 occurrences
3nd most common biome type: Tropical and subtropical dry broadleaf forest, 11 occurrences

----------- Lake Chad
Number of biomes:            2
Current biome in model:      Tropical and subtropical grassland savanna and shrubland
1st most common biome type: Tropical and subtropical grassland savanna and shrubland, 2328 occurrences
2nd most common biome type: Deserts and xeric shrubland, 277 occurrences

----------- Lake Challa
Number of biomes:            2
Current biome in model:      Tropical and subtropical grassland savanna and shrubland
1st most common biome type: Tropical and subtropical grassland savanna and shrubland, 2261 occurrences
2nd most common biome type: Tropical and subtropical coniferous forest, 344 occurrences

----------- Lake Consuelo
Number of biomes:            1
Current biome in model:      Tropical and subtropical moist broadleaf forest
Most common biome type:      Tropical and subtropical moist broadleaf forest, 2605 occurrences

----------- Lake El'gygytgyn
Number of biomes:            2
Current biome in model:      Tundra
1st most common biome type: Tundra, 2320 occurrences
2nd most common biome type: Boreal forest/taiga, 285 occurrences

----------- Lake Kopais
Number of biomes:            1
Current biome in model:      Mediterranean forest woodland and scrub
Most common biome type:      Mediterranean forest woodland and scrub, 2605 occurrences
```

```
----------- Lake Kotokel
Number of biomes:            4
Current biome in model:      Boreal forest/taiga
1st most common biome type: Boreal forest/taiga, 2362 occurrences
2nd most common biome type: Temperate conifer forest, 223 occurrences
3nd most common biome type: Tundra, 18 occurrences

----------- Lake Magadi
Number of biomes:            4
Current biome in model:      Tropical and subtropical grassland savanna and shrubland
1st most common biome type: Tropical and subtropical grassland savanna and shrubland, 1603 occurrences
2nd most common biome type: Tropical and subtropical coniferous forest, 884 occurrences
3nd most common biome type: Mediterranean forest woodland and scrub, 92 occurrences

----------- Lake Malawi
Number of biomes:            2
Current biome in model:      Tropical and subtropical grassland savanna and shrubland
1st most common biome type: Tropical and subtropical grassland savanna and shrubland, 2338 occurrences
2nd most common biome type: Tropical and subtropical moist broadleaf forest, 267 occurrences

----------- Lake Ochrid
Number of biomes:            2
Current biome in model:      Temperate conifer forest
1st most common biome type: Temperate conifer forest, 2180 occurrences
2nd most common biome type: Temperate broadleaf and mixed forest, 425 occurrences

----------- Lake Patzcuaro
Number of biomes:            3
Current biome in model:      Tropical and subtropical coniferous forest
1st most common biome type: Tropical and subtropical coniferous forest, 2273 occurrences
2nd most common biome type: Montane grassland and shrubland, 322 occurrences
3nd most common biome type: Mediterranean forest woodland and scrub, 10 occurrences

----------- Lake Petén-Itzá
Number of biomes:            1
Current biome in model:      Tropical and subtropical moist broadleaf forest
Most common biome type:      Tropical and subtropical moist broadleaf forest, 2605 occurrences

----------- Lake Prespa
Number of biomes:            4
Current biome in model:      Mediterranean forest woodland and scrub
1st most common biome type: Temperate conifer forest, 1558 occurrences
2nd most common biome type: Temperate broadleaf and mixed forest, 712 occurrences
3nd most common biome type: Mediterranean forest woodland and scrub, 329 occurrences

----------- Lake Qinghai
Number of biomes:            2
Current biome in model:      Montane grassland and shrubland
1st most common biome type: Montane grassland and shrubland, 2594 occurrences
2nd most common biome type: Temperate grassland savanna and shrubland, 11 occurrences

----------- Lake Rutundu, Mt Kenya
Number of biomes:            4
Current biome in model:      Tropical and subtropical grassland savanna and shrubland
1st most common biome type: Tropical and subtropical grassland savanna and shrubland, 2183 occurrences
2nd most common biome type: Tropical and subtropical coniferous forest, 416 occurrences
3nd most common biome type: Mediterranean forest woodland and scrub, 4 occurrences

----------- Lake Siberia 93-1
Number of biomes:            1
Current biome in model:      Tropical and subtropical moist broadleaf forest
Most common biome type:      Tropical and subtropical moist broadleaf forest, 2605 occurrences

----------- Lake Suigetsu
Number of biomes:            2
Current biome in model:      Temperate broadleaf and mixed forest
1st most common biome type: Temperate broadleaf and mixed forest, 2557 occurrences
2nd most common biome type: Tropical and subtropical moist broadleaf forest, 48 occurrences

----------- Lake Tanganyika
Number of biomes:            2
Current biome in model:      Tropical and subtropical grassland savanna and shrubland
1st most common biome type: Tropical and subtropical grassland savanna and shrubland, 2580 occurrences
2nd most common biome type: Mediterranean forest woodland and scrub, 25 occurrences

----------- Lake Tecocomulco Core TA
Number of biomes:            2
Current biome in model:      Tropical and subtropical coniferous forest
1st most common biome type: Montane grassland and shrubland, 2539 occurrences
2nd most common biome type: Tropical and subtropical coniferous forest, 66 occurrences

----------- Lake Texcoco TXB
Number of biomes:            2
Current biome in model:      Tropical and subtropical coniferous forest
1st most common biome type: Montane grassland and shrubland, 2434 occurrences
2nd most common biome type: Tropical and subtropical coniferous forest, 171 occurrences
```

```
----------- Lake Titicaca
Number of biomes:            1
Current biome in model:      Montane grassland and shrubland
Most common biome type:      Montane grassland and shrubland, 2605 occurrences

----------- Lake Tritrivakely
Number of biomes:            2
Current biome in model:      Tropical and subtropical moist broadleaf forest
1st most common biome type: Tropical and subtropical moist broadleaf forest, 1637 occurrences
2nd most common biome type: Tropical and subtropical grassland savanna and shrubland, 968 occurrences

----------- Lake Van
Number of biomes:            3
Current biome in model:      Temperate conifer forest
1st most common biome type: Temperate conifer forest, 2503 occurrences
2nd most common biome type: Temperate broadleaf and mixed forest, 81 occurrences
3nd most common biome type: Montane grassland and shrubland, 21 occurrences

----------- Lake Yamozero
Number of biomes:            5
Current biome in model:      Boreal forest/taiga
1st most common biome type: Boreal forest/taiga, 1262 occurrences
2nd most common biome type: Tundra, 1210 occurrences
3nd most common biome type: Temperate broadleaf and mixed forest, 90 occurrences

----------- Leffe Basin
Number of biomes:            1
Current biome in model:      Temperate broadleaf and mixed forest
Most common biome type:      Temperate broadleaf and mixed forest, 2605 occurrences

----------- Les Echets
Number of biomes:            2
Current biome in model:      Temperate broadleaf and mixed forest
1st most common biome type: Temperate broadleaf and mixed forest, 2553 occurrences
2nd most common biome type: Temperate conifer forest, 52 occurrences

----------- Lusitania
Number of biomes:            1
Current biome in model:      Tropical and subtropical moist broadleaf forest
Most common biome type:      Tropical and subtropical moist broadleaf forest, 2605 occurrences

----------- Maicuru
Number of biomes:            1
Current biome in model:      Tropical and subtropical moist broadleaf forest
Most common biome type:      Tropical and subtropical moist broadleaf forest, 2605 occurrences

----------- Malinche Volcan 2
Number of biomes:            3
Current biome in model:      Temperate broadleaf and mixed forest
1st most common biome type: Temperate broadleaf and mixed forest, 2542 occurrences
2nd most common biome type: Tropical and subtropical coniferous forest, 42 occurrences
3nd most common biome type: Montane grassland and shrubland, 21 occurrences

----------- Maxus Site 5
Number of biomes:            1
Current biome in model:      Tropical and subtropical moist broadleaf forest
Most common biome type:      Tropical and subtropical moist broadleaf forest, 2605 occurrences

----------- Nevado Sajama
Number of biomes:            1
Current biome in model:      Montane grassland and shrubland
Most common biome type:      Montane grassland and shrubland, 2605 occurrences

----------- Oerel
Number of biomes:            4
Current biome in model:      Temperate broadleaf and mixed forest
1st most common biome type: Temperate broadleaf and mixed forest, 1790 occurrences
2nd most common biome type: Boreal forest/taiga, 536 occurrences
3nd most common biome type: Temperate conifer forest, 216 occurrences

----------- Ogle Bridge
Number of biomes:            1
Current biome in model:      Tropical and subtropical moist broadleaf forest
Most common biome type:      Tropical and subtropical moist broadleaf forest, 2605 occurrences

----------- Padul Basin
Number of biomes:            3
Current biome in model:      Mediterranean forest woodland and scrub
1st most common biome type: Mediterranean forest woodland and scrub, 2327 occurrences
2nd most common biome type: Temperate broadleaf and mixed forest, 198 occurrences
3nd most common biome type: Temperate conifer forest, 80 occurrences
```

```
----------- Paleolake Heqing
Number of biomes:           3
Current biome in model:     Tropical and subtropical coniferous forest
1st most common biome type: Temperate broadleaf and mixed forest, 1552 occurrences
2nd most common biome type: Montane grassland and shrubland, 997 occurrences
3nd most common biome type: Tropical and subtropical coniferous forest, 56 occurrences

----------- Pantano de Genagra
Number of biomes:           2
Current biome in model:     Tropical and subtropical moist broadleaf forest
1st most common biome type: Tropical and subtropical moist broadleaf forest, 2602 occurrences
2nd most common biome type: Tropical and subtropical coniferous forest, 3 occurrences

----------- Petén Itzá
Number of biomes:           1
Current biome in model:     Tropical and subtropical moist broadleaf forest
Most common biome type:     Tropical and subtropical moist broadleaf forest, 2605 occurrences

----------- Piagua
Number of biomes:           2
Current biome in model:     Tropical and subtropical moist broadleaf forest
1st most common biome type: Tropical and subtropical moist broadleaf forest, 2602 occurrences
2nd most common biome type: Tropical and subtropical coniferous forest, 3 occurrences

----------- Pitalito PIT 11
Number of biomes:           1
Current biome in model:     Tropical and subtropical moist broadleaf forest
Most common biome type:     Tropical and subtropical moist broadleaf forest, 2605 occurrences

----------- Ponta Grossa
Number of biomes:           2
Current biome in model:     Tropical and subtropical moist broadleaf forest
1st most common biome type: Tropical and subtropical moist broadleaf forest, 2494 occurrences
2nd most common biome type: Temperate broadleaf and mixed forest, 111 occurrences

----------- Praclaux Crater
Number of biomes:           2
Current biome in model:     Temperate broadleaf and mixed forest
1st most common biome type: Temperate broadleaf and mixed forest, 1635 occurrences
2nd most common biome type: Temperate conifer forest, 970 occurrences

----------- Punta Penas
Number of biomes:           1
Current biome in model:     Temperate conifer forest
Most common biome type:     Temperate conifer forest, 2605 occurrences

----------- Ribains Crater
Number of biomes:           2
Current biome in model:     Temperate broadleaf and mixed forest
1st most common biome type: Temperate broadleaf and mixed forest, 2451 occurrences
2nd most common biome type: Temperate conifer forest, 154 occurrences

----------- Salar de Uyuni
Number of biomes:           2
Current biome in model:     Montane grassland and shrubland
1st most common biome type: Montane grassland and shrubland, 2602 occurrences
2nd most common biome type: Deserts and xeric shrubland, 3 occurrences

----------- Samerberg
Number of biomes:           4
Current biome in model:     Temperate conifer forest
1st most common biome type: Temperate broadleaf and mixed forest, 1671 occurrences
2nd most common biome type: Temperate conifer forest, 920 occurrences
3nd most common biome type: Boreal forest/taiga, 9 occurrences

----------- Serra Sul dos Carajas
Number of biomes:           2
Current biome in model:     Tropical and subtropical moist broadleaf forest
1st most common biome type: Tropical and subtropical moist broadleaf forest, 2576 occurrences
2nd most common biome type: Tropical and subtropical grassland savanna and shrubland, 29 occurrences

----------- Shudu Lake
Number of biomes:           4
Current biome in model:     Temperate conifer forest
1st most common biome type: Temperate conifer forest, 1354 occurrences
2nd most common biome type: Montane grassland and shrubland, 1010 occurrences
3nd most common biome type: Temperate broadleaf and mixed forest, 217 occurrences

----------- Stirone Section
Number of biomes:           2
Current biome in model:     Temperate broadleaf and mixed forest
1st most common biome type: Temperate broadleaf and mixed forest, 2261 occurrences
2nd most common biome type: Temperate conifer forest, 344 occurrences
```

```
----------- Stracciacappa
Number of biomes:           3
Current biome in model:     Mediterranean forest woodland and scrub
1st most common biome type: Temperate broadleaf and mixed forest, 2385 occurrences
2nd most common biome type: Mediterranean forest woodland and scrub, 216 occurrences
3nd most common biome type: Temperate conifer forest, 4 occurrences

----------- Taiquemo
Number of biomes:           1
Current biome in model:     Montane grassland and shrubland
Most common biome type:     Montane grassland and shrubland, 2605 occurrences

----------- Tarragona
Number of biomes:           2
Current biome in model:     Tropical and subtropical moist broadleaf forest
1st most common biome type: Tropical and subtropical moist broadleaf forest, 2546 occurrences
2nd most common biome type: Temperate broadleaf and mixed forest, 59 occurrences

----------- Tenaghi Philippon
Number of biomes:           4
Current biome in model:     Mediterranean forest woodland and scrub
1st most common biome type: Mediterranean forest woodland and scrub, 2576 occurrences
2nd most common biome type: Temperate grassland savanna and shrubland, 26 occurrences
3nd most common biome type: Temperate conifer forest, 2 occurrences

----------- Tswaing Crater
Number of biomes:           4
Current biome in model:     Tropical and subtropical coniferous forest
1st most common biome type: Tropical and subtropical coniferous forest, 1635 occurrences
2nd most common biome type: Montane grassland and shrubland, 893 occurrences
3nd most common biome type: Tropical and subtropical grassland savanna and shrubland, 76 occurrences

----------- Valle de Castiglione
Number of biomes:           2
Current biome in model:     Mediterranean forest woodland and scrub
1st most common biome type: Mediterranean forest woodland and scrub, 2565 occurrences
2nd most common biome type: Temperate broadleaf and mixed forest, 40 occurrences

----------- Vankervelsvlei
Number of biomes:           1
Current biome in model:     Temperate grassland savanna and shrubland
Most common biome type:     Temperate grassland savanna and shrubland, 2605 occurrences

----------- Velay Plateau
Number of biomes:           2
Current biome in model:     Temperate broadleaf and mixed forest
1st most common biome type: Temperate broadleaf and mixed forest, 2451 occurrences
2nd most common biome type: Temperate conifer forest, 154 occurrences

----------- Volta Velha
Number of biomes:           1
Current biome in model:     Tropical and subtropical moist broadleaf forest
Most common biome type:     Tropical and subtropical moist broadleaf forest, 2605 occurrences

----------- Wasa Mayu
Number of biomes:           1
Current biome in model:     Tropical and subtropical moist broadleaf forest
Most common biome type:     Tropical and subtropical moist broadleaf forest, 2605 occurrences

----------- Wonderkrater
Number of biomes:           4
Current biome in model:     Tropical and subtropical grassland savanna and shrubland
1st most common biome type: Tropical and subtropical coniferous forest, 1049 occurrences
2nd most common biome type: Montane grassland and shrubland, 998 occurrences
3nd most common biome type: Tropical and subtropical grassland savanna and shrubland, 530 occurrences

----------- Xinias
Number of biomes:           2
Current biome in model:     Mediterranean forest woodland and scrub
1st most common biome type: Mediterranean forest woodland and scrub, 2601 occurrences
2nd most common biome type: Temperate grassland savanna and shrubland, 4 occurrences

----------- Zacapu Basin
Number of biomes:           3
Current biome in model:     Tropical and subtropical coniferous forest
1st most common biome type: Tropical and subtropical coniferous forest, 2273 occurrences
2nd most common biome type: Montane grassland and shrubland, 322 occurrences
3nd most common biome type: Mediterranean forest woodland and scrub, 10 occurrences

----------- Zoige Basin
Number of biomes:           1
Current biome in model:     Montane grassland and shrubland
Most common biome type:     Montane grassland and shrubland, 2605 occurrences
```
